# Structural probing of RNA hairpins quantifies protein occupancy on RNA and links it to function in human cells

**DOI:** 10.1093/nar/gkag1019

**Published:** 2026-10-30

**Authors:** Abby R Thurm, Tanya H Nguyen, Jesus Damian Torres Campos, Matthew Mansfield, Lacramioara Bintu, Lauren D Hagler

**Affiliations:** Biophysics Program, Stanford University School of Medicine, Stanford, CA 94305, United States; Department of Chemistry, Texas A&M University, College Station, TX 77843, United States; Department of Chemistry, Texas A&M University, College Station, TX 77843, United States; Department of Chemistry, Texas A&M University, College Station, TX 77843, United States; Department of Bioengineering, Stanford University, Stanford, CA 94305, United States; Department of Chemistry, Texas A&M University, College Station, TX 77843, United States

## Abstract

RNA structures that form inside living cells govern processes ranging from translation to RNA decay, many of which are controlled by RNA-binding proteins (RBPs). Because RBP specificity can depend on both RNA sequence and folding, traditional pulldown-based methods often obscure the structural context of bound RNAs. Here, a quantitative framework based on dimethyl sulfate mutational profiling and sequencing was established to directly measure RNA structure and RBP binding in human cells at single-nucleotide resolution. Applied to a 1600-member MS2 hairpin library, this approach revealed that RNA folding is the strongest determinant of MS2 coat protein (MCP) recognition in the MS2–MCP system, requiring formation of a stable hairpin and a consensus loop sequence, with stem length or GC content contributing primarily through their effect on hairpin folding. An in-cell occupancy model was derived to quantify fractional binding from structural readouts. Complementary systems using degron-tuned MCP and a downregulatory MCP fusion demonstrated binding behavior under subsaturating protein concentrations and showed that inferred occupancy quantitatively predicts RNA degradation efficiency. Together, these results establish a quantitative strategy for measuring RBP–RNA specificity and affinity directly in cells, using the MS2–MCP system to link RNA structure, protein occupancy, and regulatory outcome.

## Graphical Abstract

**Graphical Abstract:**
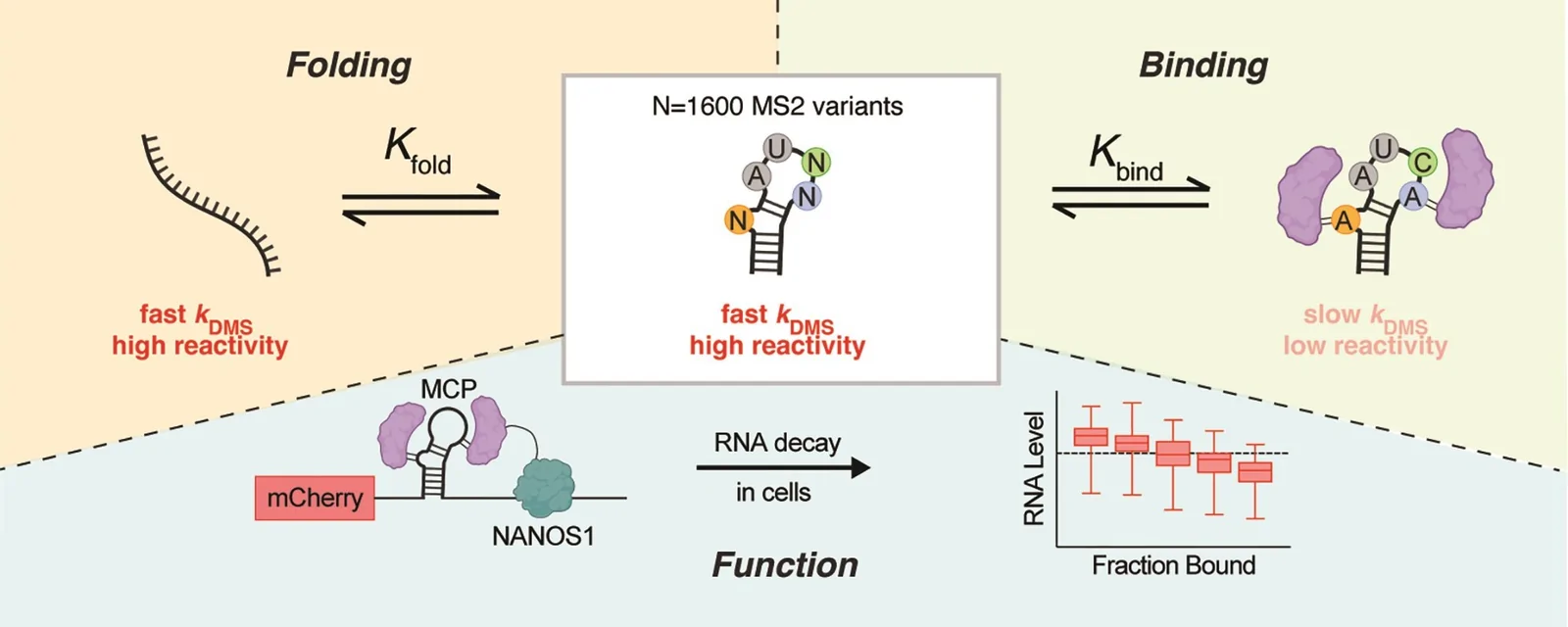

## Introduction

RNA is a central regulator of biological function that plays important roles in a wide range of cellular processes [1, 2]. By folding into dynamic secondary and tertiary structures and associating with RNA-binding proteins (RBPs), RNAs dictate complex interactions that control such fundamental processes as translation, splicing, and localization [3–7]. Importantly, RNA structure and sequence both contribute to the recognition of RNAs by RBPs, although their relative importance varies across systems. This network of interactions represents a crucial layer of post-transcriptional control that fine-tunes gene expression in response to external signals [8, 9]. In order to fully understand the extent to which RNAs regulate overall cellular behavior, it is first necessary to determine how much of a specific RNA–RNA or RNA–protein interaction, in terms of fractional stability or occupancy, is required to trigger a measurable biological function. In principle, this would involve measuring the affinity (*K*_d_) and bound fraction of an RNA–RBP complex directly in its native cellular context, where RNA sequence, structure, and protein availability are physiologically relevant. However, such measurements remain experimentally challenging with existing genomic approaches, as they require control over RNA and/or protein concentration and direct quantification of the fraction of molecules that are bound [10, 11].

High-throughput methods such as enhanced crosslinking and immunoprecipitation (eCLIP) [12] have been instrumental in identifying RNA–protein interactions across the transcriptome and in defining consensus binding motifs for RBPs [13]. By aggregating the sequences of enriched RNA fragments, eCLIP enables construction of position-weight matrices that describe sequence preferences and support inference of relative binding specificity [14]. However, these bulk measurements provide no information about the extent of binding at individual RNA sites. Because eCLIP signals reflect population-averaged enrichment across thousands of transcripts, they cannot report what fraction of a given RNA is actually bound by an RBP at endogenous levels in cells, nor how RNA structure may modulate that occupancy [15]. As a result, while eCLIP robustly identifies where RBPs can bind, it cannot determine how much binding is required to drive regulatory function at a specific RNA locus [16, 17]. Addressing this gap requires an approach that can quantify RNA–protein interactions at defined sites in living cells while simultaneously reporting on the RNA structural environment in which binding occurs.

RNA chemical probing has been traditionally used to predict the secondary structure of RNAs transcriptome-wide [18]. Methods like DMS-MaPseq (dimethyl sulfate mutational profiling with sequencing) [19] and SHAPE (selective 2′-hydroxyl acylation analyzed by primer extension) [20] use chemical probes like dimethyl sulfate and anhydrides or imidazolides, respectively, to selectively modify RNA based on its structural state. During DMS-MaPseq, dimethyl sulfate is used to methylate the Watson-Crick-Franklin face of adenine (N1) and cytosine (N3) nucleotides when they are unpaired and solvent-exposed, enabling discrimination between structured and unstructured regions of RNA [21]. Methylations are converted to mutations during reverse transcription and detected by next-generation sequencing [22, 23]. Mutational profiles can be used to constrain secondary structure prediction and discover novel RNA structures, often with functional consequences [18]. Despite its power, RNA chemical probing has largely been applied to determine where RNA is folded, rather than how much of an RNA population occupies a given structural state or how that stability relates to downstream function [24]. The contribution of RNA structure to RBP recognition varies considerably across RBPs, ranging from interactions that depend on well-defined structural motifs to those driven primarily by sequence, with structure playing a more modulatory role [25, 26]. Existing applications of DMS-MaPseq, however, do not explicitly relate RNA structural ensembles to RBP occupancy in living cells, regardless of the underlying mode of recognition. This limitation motivates the development of approaches that leverage chemical probing not only to report RNA structure, but also to quantitatively measure RNA–protein interactions in their native cellular context.

The differential reactivity of dimethyl sulfate (DMS) based on the local environment of the RNA is not limited to constraining models for structure prediction; it can also be used to map binding events of other RNAs, proteins, and small molecule ligands by detecting changes in RNA accessibility due to direct shielding or a change in conformation [21, 27–33]. Building upon this principle, we developed a quantitative framework for measuring structural stability and occupancy simultaneously in human cells using RNA chemical probing. A systematic library in which each position of an RNA is individually mutated enabled direct assessment of how each single-nucleotide change influences local folding, protein binding, and downstream functional outcomes. To develop and validate this framework, we selected the well-characterized MS2 hairpin–MS2 coat protein (MCP) interaction as a model system because its structural determinants of binding are well defined and the MCP-contacting bases are known (Fig. 1A) [34–36]. DMS-MaPseq can effectively and sensitively measure MCP binding to MS2 hairpins in cells, confirming previous data showing that stable hairpin formation is a prerequisite for MCP binding and that single-nucleotide changes in the loop can quantitatively modulate affinity. Incorporation of a degron-controlled MCP variant further enabled modulation of intracellular protein levels to values similar to those of endogenous RBPs, allowing characterization of binding preferences under subsaturating conditions. Finally, a quantitative framework for MCP occupancy in living cells showed that these predictions correlate with the functional output of a downregulatory RBP, establishing a formative link between occupancy and function through a base-resolved method for measuring RBP binding in cells.

**Figure 1.**
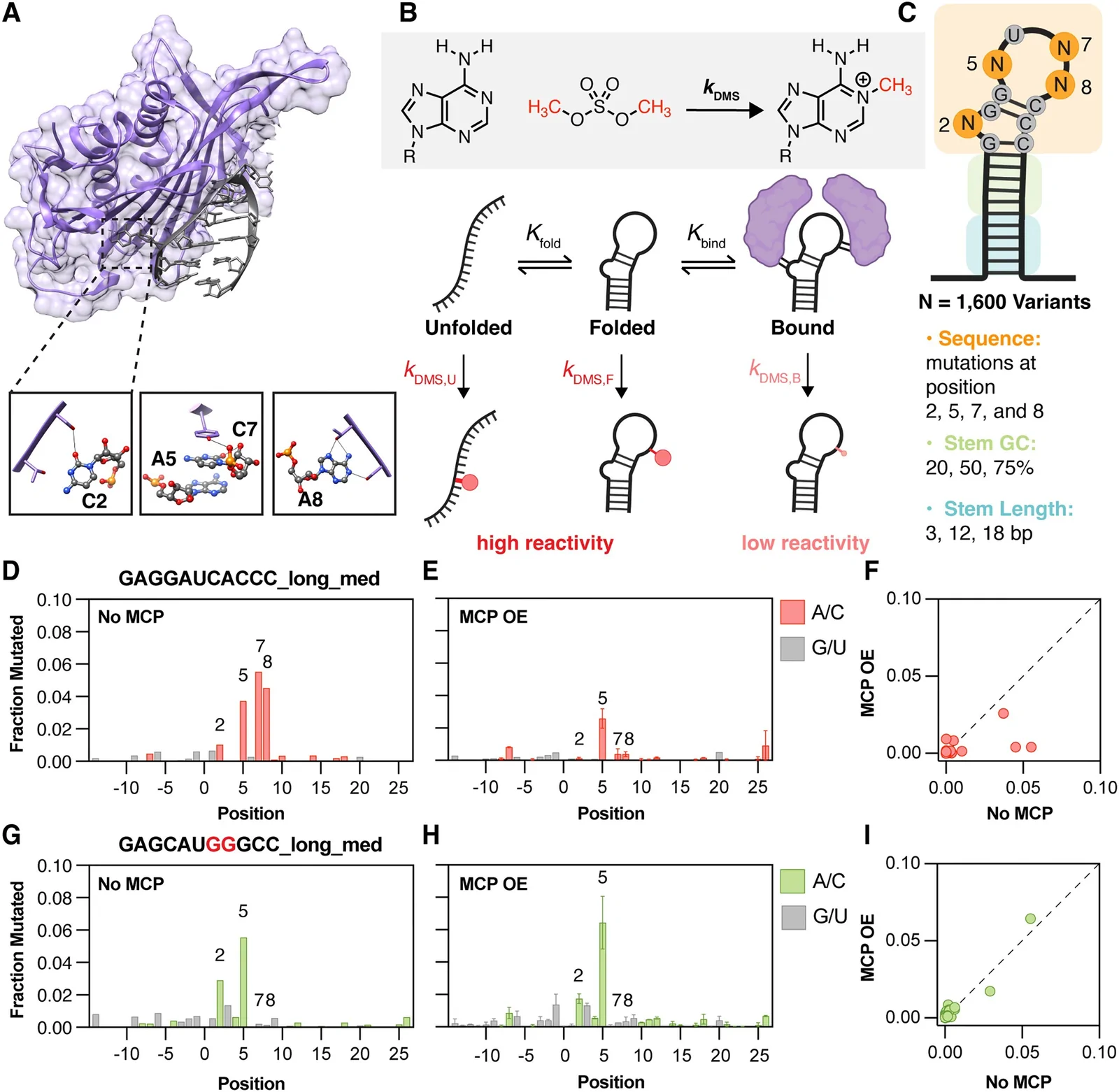
DMS-MaPseq measurements of RNA–protein binding. (**A**) Top, X-ray structure of MCP (purple) recognizing and binding to the canonical MS2 stem–loop (gray, PDB: 2IZM) [44]. Bottom, specific MCP residues make direct contact with designated places on the MS2 hairpin. Tyrosine 85 touches the first and third positions of the tetraloop (5 and 7 by our numbering scheme), serine 47 touches the bulge nucleotide at position 2, and threonine 45/serine 47 make contact with the fourth position of the tetraloop (A8). (**B**) Thermodynamic scheme for RNA folding and binding. Kinetic scheme for modification by DMS, which methylates the N1 position of adenine or the N3 position of cytosine bases. We assume that RNA can exist in three states: unfolded, folded, and folded+bound (bound). The rate of DMS modification at accessible (unpaired) bases is relatively high, leading to high DMS signal under those conditions. When the RNA is completely bound by MCP, the rate of DMS modification at unpaired but bound positions is very low or effectively 0. (**C**) A 1 600-member MS2 stem-loop library varying base identity at positions 2, 7, and 8, stem length, and stem GC content was cloned into the 3′UTR of an mCherry-expressing base plasmid. DMS reactivity (fraction mutated) at all positions of a wild-type MS2 stem-loop GAGGAUCACCC in the absence (**D**) and presence (**E**) of MCP. A and C bases are represented by red bars. G and U bases are represented by gray bars. Error bars represent the range of two independent replicates. (**F**) Comparison of DMS-MaPseq reactivity at the same positions in the absence (*x*-axis) and presence (*y*-axis) of MCP, for the same wild-type stem–loop in panels (D) and (E). Each red circle represents one base averaged over two independent replicates. The line of identity (*y* = *x*) is represented by a dashed line. DMS reactivity (fraction mutated) at all positions of a mutated MS2 stem–loop GAGCAUGGGCC in the absence (**G**) and presence (**H**) of MCP. A and C bases are represented by green bars. G and U bases are represented by gray bars. Error bars represent the range of two independent replicates. (**I**) Comparison of DMS-MaPseq signal at the same positions in the absence (*x*-axis) and presence (*y*-axis) of MCP, for the same mutant stem–loop in panels (G) and (H). Each green circle represents one base averaged over two independent replicates. The line of identity (*y* = *x*) is represented by a dashed line.

## Materials and methods

### Cell lines and cell culture

All experiments were carried out in HEK293T-LentiX (Takara Bio, 632180, female), which were cultured in a controlled humidified incubator at 37°C and *5%* CO2 in DMEM (Gibco, 10569069) media supplemented with *10%* FBS (Omega Scientific, 20014T) and *1%* Penicillin-Streptomycin-Glutamine (Gibco, 10378016). This cell line was not authenticated. All cell lines tested negative for mycoplasma.

### Generation of stable DHFR-MCP- and DHFR-MCP-RRE-expressing cells

Lentiviral production was performed by seeding HEK293T Lenti-X cells at 5 × 10^5^ cells per well in 2 ml of DMEM in six wells of a six-well plate. After 24 h, cells were transfected with 750 ng of an equimolar mixture of three third-generation production plasmids (pMD2.G: Addgene #12259; pRSV-Rev: Addgene #12253; pMDLg/pRRE: Addgene #12251; all gifts from D. Trono) and 750 ng of pAT124 (DHFR-MCP overexpression plasmid). The four plasmids were incubated for 15 min with 5 μl of polyethylenimine (PEI, Polysciences #23966) before transfection. After 72 h of incubation, lentivirus was harvested and collected, and supernatant was filtered through 0.45-μm PES filters (CELLTREAT #229749). Twelve milliliters of virus was concentrated into 1 ml using Pierce PES Protein Concentrators (Thermo Fisher #88533), which was added directly to a 10-cm plate of HEK293T cells at *50%* confluence. After 48 h of culture, antibiotic selection was initiated with blasticidin (10 μg/ml, Gibco #A1113903) and infection and selection efficiency were monitored daily with flow cytometry on a Bio-Rad ZE5 Cell Analyzer (Bio-Rad #12004278).

A second stable line expressing DHFR-MCP-RRE was generated in parallel by the same procedure, substituting pAT128 (DHFR-MCP-RRE overexpression plasmid) for pAT124 during lentiviral production. Both lines were selected and monitored identically.

### MS2 stem-loop library design

Three base wild-type MS2 stem-loops were used for successive mutation: GAGGAUCACCC, CACCAUCAGGG, and GAGCAUCAGCC. All possible single, double, and triple substitutions of the four nucleotides (A,G,C,U) were created for each stem-loop at positions A2 (bulge), C7 (third tetraloop), and A8 (fourth tetraloop) for 192 total stem-loops. Several presumed non-binding sequences were also created by taking the base stem-loops GAGGAUUACCC, CACCAUUAGGG, and GAGCAUUAGCC and substituting the conserved position A5 (first tetraloop) for C, G, and U. Two sequences with the bottom C-G stem of the stem–loop disrupted, AGAGAUUACCU and GGAAAUUAUCC, were also added as non-binding controls for a total of 206 base stem-loops.

Six stem variants were designed: 9- and 15-bp long with low (*20%*), medium (*50%*), or high (*75%*) GC content. Each loop was added to each stem variant, plus repeated once with no additional stem past the 3 G-C pairs in the loop, for a total of 1442 members. Finally, a set of 16 stem-loops (the consensus GAGGAUCACCC plus all variants at the 7th and 8th positions) added to a progressively longer stem of 1-10 base pairs for a total of 160 additional sequences (“Parent-child” library members) were created for a total of 1602 library members.

The final library sequences were constructed as follows: a forward primer-binding sequence for amplification (TTAAACCGGCCAACATACC), a C/T shuffled buffer sequence, the forward stem sequence, the loop variant, the reverse stem sequence, another C/T shuffled buffer sequence, a unique 15-nucleotide barcode sequence (no closer than Hamming distance of 3 to any other barcode), and a reverse primer-binding sequence CGCTACTCGTTCCTTTCGA. The C/T buffer sequences on each side of the stem–loop were variable in length so that each library member was exactly 170 nucleotides long. The library was ordered as a pool from Twist Biosciences. The sequence for each member of the library can be found in Supplementary Table S1.

### Pooled library cloning

The Twist oligo pool was resuspended to 10 ng/μl in water and the library was PCR (polymerase chain reaction) amplified using primers specific to the appended primer-binding sequences, above. The reactions were prepared in a pre-PCR hood to reduce contamination. PCR was performed using 10 μl Q5 polymerase buffer (NEB #B9027S), 10 μl High GC Enhancer (NEB #B9028A), 1 μl dNTP mix (NEB #N0447S), 0.5 μl Q5 polymerase (NEB #M0491S), 2.5 μl each of forward and reverse primer (10 μM), and 1 μl of the library pool (10 ng). The following protocol was used: initial denaturation at 98°C for 30 s; six cycles of 98°C for 10 s, 63°C for 30 s, and 72°C for 60 s; and final extension at 72°C for 2 min. The amplified library was purified with 0.9X SPRISelect (Beckman Coulter #B23317) and elution in 20 μl.

pcDNA3.1-mCherry (Addgene #128744) was double-digested with 10 000 U/ml NotI-HF (NEB #R3189L) and XbaI (NEB #R0145L) for 30 min at 37°C, using 1 μl of each enzyme per 5 μg plasmid. After heat inactivation at 65°C for 20 min, the digested plasmid was run on a *0.5%* TAE gel until a linearized band could be extracted using the QIAquick Gel Extraction Kit (Qiagen #28704). The amplified library was cloned into the digested pcDNA3.1 using Gibson assembly with the NEBuilder HiFi DNA Assembly kit (NEB #E5520S) as follows: eight Gibson reactions were prepared using 10 μl HiFi kit, 15 ng of PCR product, 100 ng of vector, and nuclease-free water to 20 μl. Reactions were incubated at 50°C for 1 h, then pooled together and concentrated to 6 μl using the Zymo Clean&Concentrate DNA kit (Zymo #D4004).

2.5 μl of the pooled Gibson assemblies were electroporated into 50 μl of Endura DUO electrocompetent cells (Lucigen #60242-2) using Gene Pulse Electroporation Cuvettes with a 0.1-cm band gap (Bio-Rad #1652089) and a Gene Pulser Xcell Total System (Bio-Rad #1652660) under the following conditions: 1.8 kV, 10 μF, 600 Ω, and 0.1 cm distance. Cells were recovered in 2 ml of 37°C SOC recovery medium (NEB #B9020S) at 37°C for 1 h, after which they were added directly to 500 ml LB with 100 μg/ml carbenicillin. Cells were incubated overnight at 30°C, after which the plasmid pool was extracted using the Qiagen Plasmid Maxi Kit (Qiagen #12162) and library quality was assessed using Illumina sequencing after PCR amplification from the plasmid pool.

### Transfection of stem-loop library and MCP constructs

Libraries and overexpression plasmids were transfected into wild-type or DHFR-MCP-expressing HEK293T cells using Lipofectamine 3000 following the manufacturer’s protocol. Briefly, one 10-cm plate for each biological replicate and experimental condition was seeded with 4 × 10^6^ HEK293T cells. Twenty-four hours later, 7 μg of library alone or 7 μg of library plus 7 μg of pAT031 (MCP overexpression plasmid) or pAT121 (MCP–RRE degrader plasmid) were transfected onto each plate using 21.7 μl of Lipofectamine 3000 and 28 μl of P3000 reagent. After 48 h, cells were processed either for DMS-MaPseq or RNA-seq (see below). For experiments with stable DHFR-MCP- or DHFR-MCP-RRE-expressing HEK293T cells, trimethoprim (TMP) was added to a given concentration (0, 0.1, 1, or 10 μM final) 24 h before library transfection to allow for MCP expression, then refreshed every 24 h with media changes to maintain constant drug concentration through the course of the experiment.

### DMS-MaPseq for detection of RNA structure and MCP binding

Ten milliliters of DMEM per 10-cm plate of HEK293T cells was warmed in preparation for DMS modification. Ten milliliters per sample of each of *30%* 2-mercaptoethanol (Sigma–Aldrich #M6250) in phosphate buffered saline (PBS; Thermo Fisher #14190250), *10%* 2-mercaptoethanol in PBS, and PBS alone were chilled on ice in a chemical fume hood. Cells in 10-cm plates were brought into a tissue culture hood and media was aspirated. Two percent (v/v) of DMS (Thermo Fisher #430831000) was added to 10 ml DMEM per sample using a needle or under hypoxic conditions, quickly vortexed, and immediately added to cells. Cells were returned to a 37°C incubator for exactly 3 min, after which they were brought to the chemical fume hood and quenched with 10 ml per sample of ice-cold *30%* 2-mercaptoethanol in PBS. After quenching, cells were moved to a 50-ml conical tube and centrifuged for 5 min at 1000 × *g* at 4°C. Pellets were resuspended in ice-cold *10%* 2-mercaptoethanol in PBS and re-centrifuged for 5 min at 1000 × *g* at 4°C. Finally, pellets were resuspended in ice-cold PBS and centrifuged for 5 min at 1000 × *g* at 4°C, after which they were resuspended in 1 ml Trizol (Thermo Fisher #15596026) and frozen at −80°C.

In cell DMS-MaPseq experiments were also performed under conditions varying the DMS concentration 0.5–5% (v/v) for 3 min and varying the reaction time 0.5–10 min at *2%* (v/v), following the same procedure as above. Sample information for each experiment can be found in Supplementary Table S2.

### Targeted RNA-seq for detection of stem-loop library member abundances

The stem-loop library was expressed for 48 h in wild-type HEK293T cells together with either pAT031 (MCP overexpression plasmid) or pAT121 (MCP–RRE overexpression plasmid), or in stable DHFR-MCP-RRE-expressing cells across a TMP titration (0, 0.1, 1, and 10 μM), after which each 10-cm plate was harvested by centrifugation. Library prep was performed as follows with DMS samples, below.

### RNA library preparation for Illumina sequencing for DMS-MaPseq and RNA-seq samples

RNA was extracted using Trizol-chloroform extraction as follows: 200 μl chloroform (VWR #J67241-AP) was added to each Trizol sample, vortexed well, and centrifuged for 15 min at 15 000 × *g*. The upper (aqueous) layer was mixed with an equal volume of *70%* ethanol, then added to an RNEasy column from the RNEasy Mini kit (Qiagen #74106). RNA was extracted with the kit by following the rest of the protocol per manufacturer’s instructions. Five micrograms of each RNA sample was depleted of rRNA species by incubation with 5 μg subtraction oligos (IDT, sequences ordered from Adiconis *et al*.) [37] in a total volume of 1× hybridization buffer (200 mM NaCl, 100 mM Tris, pH 7.5) for 1 min at 68°C, then the temperature ramped down at a rate of 1°C/min to 50°C. Ten microliters RNase H buffer and 1 μl thermostable RNase H (NEB #M0523S) were added directly to each reaction along with 59 μl nuclease-free water. Reactions were incubated for 20 min at 50°C, then purified using the Zymo RNA Clean&Concentrate-5 kit (Zymo #R1013) with pre-column DNase I treatment as included in the kit.

One microgram of each rRNA-depleted RNA sample was then diluted to 11 μl in nuclease-free water and incubated with 1 μl of 10 μM reverse primer, designed to bind the reverse primer-binding sites on the library members, and 1 μl dNTP solution mix (10 mM, NEB #N0447S) for 5 min at 65°C. Four microliters Induro RT reaction buffer (NEB #B0681), 0.5 μl RNase inhibitor (NEB #M0314), 1 μl Induro Reverse Transcriptase (NEB #M0681S), and 1.5 μl nuclease-free water were added to each reaction, which were then incubated for 90 min at 55°C. Following incubation, 1 μl of 4 M NaOH was added to each reaction and incubated for 3 min at 95°C to quench reverse transcription. The resultant single-stranded DNA samples were purified using the Zymo DNA Clean&Concentrate-5 kit (Zymo #D4004) following the protocol for ssDNA.

Samples were amplified with PCR with the following protocol: 10 μl high GC enhancer, 0.5 μl Q5 polymerase, 1 μl dNTP mix, 10 μl Q5 buffer, 2.5 μl each of 10 μM forward and reverse primers that bind the sites designed on the library members, 3 μl each cDNA product, and 20.5 μl nuclease-free water. They were amplified as follows: initial denaturation at 98°C for 30 s; 25 cycles of 98°C for 10 s, 63°C for 30 s, and 72°C for 60 s; and final extension at 72°C for 2 min. Samples were purified with the Zymo DNA Clean&Concentrate-5 kit and eluted in 10 μl nuclease-free water. Two-hundred fifty nanograms of purified dsDNA product was prepared for Illumina sequencing following the NEBNext Ultra II DNA Library Prep Kit for Illumina (NEB #E7645L) following manufacturer’s instructions. Briefly, 250 ng of DNA was diluted to 50 μl and incubated with DNA End Prep Enzyme Mix and Buffer (#E7646) for 30 min at 20°C followed by 30 min at 65°C, then added to 2.5 μl undiluted Illumina adaptor, Ligation Master Mix (#E7648), and Ligation Enhancer (#E7374) for 15 min at 20°C. USER enzyme was used to digest closed-loop adapters for 15 min at 37°C, and reactions were purified with a double-sided 0.5× followed by 0.25× SPRISelect purification. Finally, samples were amplified with Q5 Ultra II Master Mix (#E7649) using NEBNext Multiplex Oligos for Illumina (NEB #E7730) before final purification with 0.9× SPRISelect cleanup. Libraries were quantified using the Qubit dsDNA HS Assay Kit (Thermo #Q33231) on a Qubit 4 Fluorometer (Fisher #Q33238) and assessed for purity on an Agilent TapeStation (Agilent #G2964AA) before being sequenced on an Illumina NovaSeq with 2 × 150 cycles.

### DMS-MaPseq data analysis

The FASTQ files were processed using SEISMIC-RNA (version 0.24.2) [28] as previously described [29]. Briefly, the function *wf* (for workflow) was executed including *-x* for two separate FASTQ files of paired-end reads and a reference FASTA file containing the sequence of each reference in the library. Processed data were further analyzed and visualized using custom Python scripts. The data were replotted in GraphPad Prism 10 to increase the resolution for publication.

### RNA-seq data analysis

Processing scripts were modified from https://github.com/bintulab/Viral_Ludwig_2022/tree/main/final_April-2023/RNA-seq%20Scripts. Briefly, reads were demultiplexed with bcl2fastq and aligned to a reference transcriptome with hisat2. The reference transcriptome was built from a .fasta file containing the sequences of all MS2 stem–loop library members using hisat2-build. Output SAM files were converted to BAM files, sorted, and indexed using samtools. Differential expression analysis was performed in R with the Bioconductor DESeq2 package [38] and a set of custom R scripts, based on the following tutorial: http://bioconductor.org/help/course-materials/2016/CSAMA/lab-3-rnaseq/rnaseq_gene_CSAMA2016.pdf. Further analyses were performed and visualized using custom Python scripts.

### Normalized difference between conditions

To quantify the magnitude of per-nucleotide changes in mutational signal between two experimental conditions, a normalized difference (Norm. Diff.) was computed at each position *j*:


\begin{eqnarray*}
\mathrm{Norm}.\, \mathrm{Diff.} \left( {{{M}_{1,j}}, {{M}_{2,j}}} \right) = \frac{{\left| {{{M}_{1,j}} - {{M}_{2,j}}} \right|}}{{\sqrt {{{M}_{1,j}} + {{M}_{2,j}}} }},
\end{eqnarray*}

where *M*_1,*j*_ and *M*_2,*j*_ are the per-nucleotide mutation fractions at position *j* in the two conditions being compared. The numerator is the absolute change in mutation fraction between conditions. The denominator applies a square-root, variance-stabilizing transform motivated by the counting noise of mutational profiling. Because the variance of a mutation measurement scales with its mean [39], dividing by the square root of the sum rescales each change by the characteristic magnitude of its sampling noise, so that positions of differing reactivity can be compared on a common scale.

Significance thresholds were established empirically before conditions were compared. Norm. Diff. was first computed between replicate samples of the same condition, where no true between-condition differences should exist, to obtain the per-position distribution of values expected from sampling and biological noise alone. The 95th percentile of this replicate distribution was used as the significance threshold. Norm. Diff. was then computed between conditions, and a position was called significantly changed when its value exceeded this threshold. Because this metric is defined on the absolute difference, this comparison constitutes a two-sided test at *α* = 0.05, capturing changes in either direction.

### Fitting of DMS reactivity to predicted folding stability

Reactivity and binding data were modeled as two-state transitions between folded and unfolded hairpin. Assuming a Boltzmann distribution over the two states, the population of each state follows a sigmoid in folding stability. Because the measured quantities are bounded by variables of the fully folded and fully unfolded endpoints rather than by 0 and 1, the population term was scaled by an amplitude *a* and offset by *a* baseline *c*. DMS reactivity (Fig. 2G) was fitted to


\begin{eqnarray*}
y = \frac{a}{{1 + b{{\mathrm{ e}}^{\left( { - \frac{x}{{\mathrm{ RT}}}} \right)}}}} + c,
\end{eqnarray*}

where *y* is the mutation fraction of each position (C9 in Fig. 2G), *x* is the mFold-predicted ∆*G*_fold_, RT = 0.617 kcal/mol at 37°C, *c* is the reactivity of the fully folded state, *a *+ *c* is the reactivity of the fully unfolded state, and *b* is a fitted parameter setting the ∆*G*_fold_ at which the transition occurs.

Binding data (Fig. 2H) were fitted to the complementary form of the same expression,


\begin{eqnarray*}
y = a\left( {\frac{{b{{\mathrm{ e}}^{\left( { - \frac{x}{{\mathrm{ RT}}}} \right)}}}}{{1 + \left( {b{{\mathrm{ e}}^{\left( { - \frac{x}{{\mathrm{ RT}}}} \right)}}} \right)}}} \right) + c,
\end{eqnarray*}

where *y* is the normalized difference averaged across MCP-contacting positions, *c* is the value in the unbound limit, and *a *+ *c* is the value at saturation. In both cases RT was fixed and *a, b*, and *c* were fitted by nonlinear regression in GraphPad Prism.

### Derivation of fraction bound from DMS-MaPseq mutation data

The mutation fraction at each position *j* as measured by DMS-MaPseq for a given RNA sequence can be represented by the initial rate equation.


\begin{eqnarray*}
\mathrm{ M}{{\mathrm{ F}}_j} \propto \mathop \sum \limits_{i = 1}^N ({{P}_{i }}{{k}_{i,j}}) \bullet \left[ {\mathrm{ DMS}} \right]t,
\end{eqnarray*}

where *P*_*i*_ is the probability of state *i, k*_*i,j*_ is the rate constant of the DMS reaction of base *j* in state *i*, and [DMS]*t* is the concentration of DMS and time of the reaction, respectively. This model is agnostic to the number of states *N*: an ensemble of arbitrary complexity contributes to the observed mutation fraction as a population-weighted average of per-state reactivities, and the form of the equation is unchanged regardless of how many conformers are populated. For the library described here, we take *N *= 3—unfolded, folded, and unbound, and MCP-bound—a simplification justified by the simple hairpin design of the constructs. We emphasize that this choice affects the interpretation of the fitted populations rather than the validity of the model. Were additional folded conformers present, they would be absorbed into *P*_folded_ as a weighted average, and the reported quantity would represent the total population of DMS-accessible, unbound conformers rather than a single defined structure.

The DMS modification of the unfolded and folded RNA states (while unbound) follows second-order rate kinetics, in which the reaction is dependent both on the number of RNA molecules existing in that state and on the concentration of DMS. In the bound RNA state, the modification rate is considered negligible due to shielding by MCP, which reduces the pseudo-second-order rate constant to near zero (pseudo-second order reaction, *k*_DMS, *B, j*_ = 0).

The observed mutation fraction in each experiment for a given position is thus


\begin{eqnarray*}
{\mathrm{MF}}_{j,\mathrm{observed}} &=& \big({P}_{\mathrm{unfolded}}{k}_{\mathrm{ DMS},U,j} [ {\mathrm{ DMS}}] + {P}_{\mathrm{folded}} {k}_{\mathrm{ DMS},F,j} [ {\mathrm{ DMS}}]\\&& +\, {P}_{\mathrm{bound}} {k}_{\mathrm{ DMS},B,j}[ {\mathrm{ DMS}}] \big)t.
\end{eqnarray*}

Using the stem length and folding analyses in Fig. 2, we have determined that every library member with a medium or long stem is folded in cells, evidenced by a lack of DMS reactivity at the paired stem bases. By restricting our analyses to only those library members, we know that the fraction of unfolded RNAs is negligible (*P*_unfolded_≈ 0). We also use only RNA positions that directly contact MCP to approximate binding, for which the rate of DMS reactivity (*k*_DMS,B_) is significantly decreased when bound to MCP. We can then simplify


\begin{eqnarray*}
\mathrm{ MF}_{j, \mathrm{observed}}^{ } = {{P}_{\mathrm{folded}}}{{k}_{\mathrm{ DMS}, F, j}}\left[ {\mathrm{ DMS}} \right]t.
\end{eqnarray*}

We also assume that all measured positions in the absence of MCP can be represented by *P*_folded_ = 1 (since we selected library members that fold completely), which means that


\begin{eqnarray*}
\mathrm{ MF}_{j, \mathrm{observed}}^{ - \mathrm{ MCP}} = {{k}_{\mathrm{ DMS}, F, j}}\left[ {\mathrm{ DMS}} \right]t.
\end{eqnarray*}

This indicates that each mutation fraction that we measure at a binding position is directly related to the probability of that sequence existing in a folded, but not bound, state. We solve for *P*_folded_:


\begin{eqnarray*}
{{P}_{\mathrm{folded}}} = \frac{{M\mathrm{ {{F}}_{j, \mathrm{observed}}}}}{{{{k}_{\mathrm{ DMS},F,j}} \left[ {\mathrm{ DMS}} \right]t}}.
\end{eqnarray*}

When the sequence is completely folded and bound by MCP, *P*_folded_ = 0; inversely, *P*_folded_ = 1 indicates a complete lack of protein binding at that position. We assume that the wild-type sequence GAGGAUCACCC_long_med, shown in Fig. 1, is completely bound and assign it as a reference wild-type sequence. We can then calculate a change in abundance between the folded and unbound fraction for a mutant RNA compared to the WT, *ΔP*_folded_:


\begin{equation*}{\mathrm{\Delta }}{{P}_{\mathrm{folded}}} = P_{\mathrm{folded}}^{\mathrm{ mut}} - P_{\mathrm{folded}}^{\mathrm{ WT}} = \frac{{\mathrm{ MF}_{j, \mathrm{observed}}^{\mathrm{ mut}}}}{{{{k}_{\mathrm{ DMS},F,j}} \left[ {\mathrm{ DMS}} \right]t}} - \frac{{\mathrm{ MF}_{j, \mathrm{observed}}^{\mathrm{ WT}}}}{{{{k}_{\mathrm{ DMS},F,j}} \left[ {\mathrm{ DMS}} \right]t}}.\end{equation*}

We remove all sequences with *ΔP*_folded_ > 1, as these are not physically possible values and represent measurement noise. Importantly, we also reduce our analysis to only common bases shared between the reference wild-type and each mutant sequence to ensure a constant k_DMS_, which is variable per base, although this removes all sequences in the library that are mutated at all of positions 2, 7, and 8.

This leaves us with a final simplification of


\begin{eqnarray*}
{\mathrm{\Delta }}{{P}_{\mathrm{folded}}} = \frac{{\mathrm{ MF}_{j,\mathrm{observed}}^{\mathrm{ mut}, + \mathrm{ MCP}}}}{{\mathrm{ MF}_{j,\mathrm{observed}}^{\mathrm{ mut}, - \mathrm{ MCP}}}} - \frac{{\mathrm{ MF}_{j,\mathrm{observed}}^{\mathrm{ WT}, + \mathrm{ MCP}}}}{{\mathrm{ MF}_{j,\mathrm{observed}}^{\mathrm{ WT}, - \mathrm{ MCP}}}}.
\end{eqnarray*}

Since we assume these RNAs are entirely folded, the fraction bound is simply


\begin{eqnarray*}
{{f}_{\mathrm{bound}}} = {\mathrm{\Delta }}{{P}_{\mathrm{bound}}} = 1 - {\mathrm{\Delta }}{{P}_{\mathrm{folded}}}.
\end{eqnarray*}

## Results

### A systematic MS2 hairpin library for quantitative analysis of MCP binding in human cells

To establish a method for systematically quantifying RNA–protein binding with single-nucleotide sensitivity while simultaneously measuring RNA structure, a systematic library was generated utilizing the well-studied MS2 hairpin RNA sequence and its cognate protein binder MCP, which are derived from the MCP bacteriophage and are orthogonal to endogenous RNA–protein interactions in human cells [40, 41]. Furthermore, MCP has successfully been used in the past to identify MS2 sequence determinants of affinity, association, and dissociation rates, and alternative RNA structures *in vitro* [36]. A library of 1600 MS2 hairpin mutants was designed based on the wild-type consensus sequenceGAGGAU(C/U)ACCC to dissect the sensitivity of using DMS to detect changes in MCP binding probabilities (Supplementary Table S1). The RNA sequence was systematically varied to modulate each of the contributing thermodynamic and kinetic parameters (*K_f_*_old_: probability of RNA folding, *K*_bind_: probability of protein binding, and *k*_DMS_: rate of DMS modification) based on the stability of the resulting RNA structure and MCP binding affinity (Fig. 1B). Initially, a wild-type MS2 stem–loop [42] (GAGGAUCACCC) was inserted in a variable stem region in the 3′UTR of a coding region for the fluorescent protein mCherry (Fig. 1C). To modulate *K*_bind_, all possible base mutants of the bulge A (A2), the C in the third position of the tetraloop (C7), and the A in the fourth position of the tetraloop (A8) were included, while the first A and U in the tetraloop were left unchanged. To vary *K*_fold_, the stem region surrounding the wild-type stem-loop was systematically varied to include 3, 12, or 18 base-pairs; three variants of different GC content (30%, 50%, or 80%) were designed for each of the longer two stems. Additionally, the directionality of the GC base-pairs that comprise the canonical minimal stem were also systematically flipped to CG to investigate how sensitive MCP binding is to base-pair identity. The entire library was transfected as a plasmid pool into HEK-293T cells either alone or co-transfected with a plasmid coding for MCP. DMS-MaPseq chemical probing was subsequently performed on each sample and the modified RNAs were sequenced and analyzed using SEISMIC-RNA [28, 29].

All 1580 library members were recovered after applying stringent quality metrics (Supplementary data), and we examined the fraction of sequencing reads that showed DMS-induced mutations at each A or C nucleotide position for each different MS2 sequence. The recovered library contained 203 short-stem variants, 608 medium-stem variants, 609 long-stem variants, and 160 “parent-child” variants used for the systematic analyses of stem length and mutation effects shown in Fig. 2. The raw mutation fractions were very low in non-DMS-treated samples, confirming that called mutations are due to DMS modification and not to sequence variability (Supplementary Fig. S1A). The mutation fractions between DMS-treated MCP overexpression replicates were highly reproducible (Supplementary Fig. S1B, *r* = 0.91). The correlation between the mutation fractions of samples with and without MCP overexpression was significantly lower than between replicates (*r* = 0.74), signifying a difference in measured DMS signal upon MCP overexpression and RNA binding (Supplementary Fig. S1C). The mutation fraction increased with both DMS concentration (Supplementary Fig. S2A and B) and reaction time (Supplementary Fig. S2C and D) up to and past our working conditions of *2%* (v/v) DMS and 3 min, saturating only at higher doses and distorting only at the longest time. In contrast, at MCP-contacted positions, the mutation fraction is significantly reduced and remained low and largely unchanged across DMS concentration and reaction time in the presence of MCP (Supplementary Fig. S2E). This observation indicates that under our conditions, DMS modification reports on the underlying ensemble of RNA states rather than perturbing RNA structure or MCP binding, as has been previously shown [43]. A normalized difference was computed for each position from the DMS mutation fractions within replicates and with and without MCP overexpression [Equation (1)]:


(1) \begin{eqnarray*}
\mathrm{Norm}.\,{\mathrm{ }}\mathrm{Diff.}{\mathrm{ }}\left( {{{M}_1},{\mathrm{ }}{{M}_2}} \right) = \frac{{\left| {{{M}_1} - {{M}_2}} \right|}}{{\sqrt {{{M}_1} + {{M}_2}} }},
\end{eqnarray*}

where *M*1 = mutation fraction of condition 1 and *M*2 = mutation fraction of condition 2.

**Figure 2.**
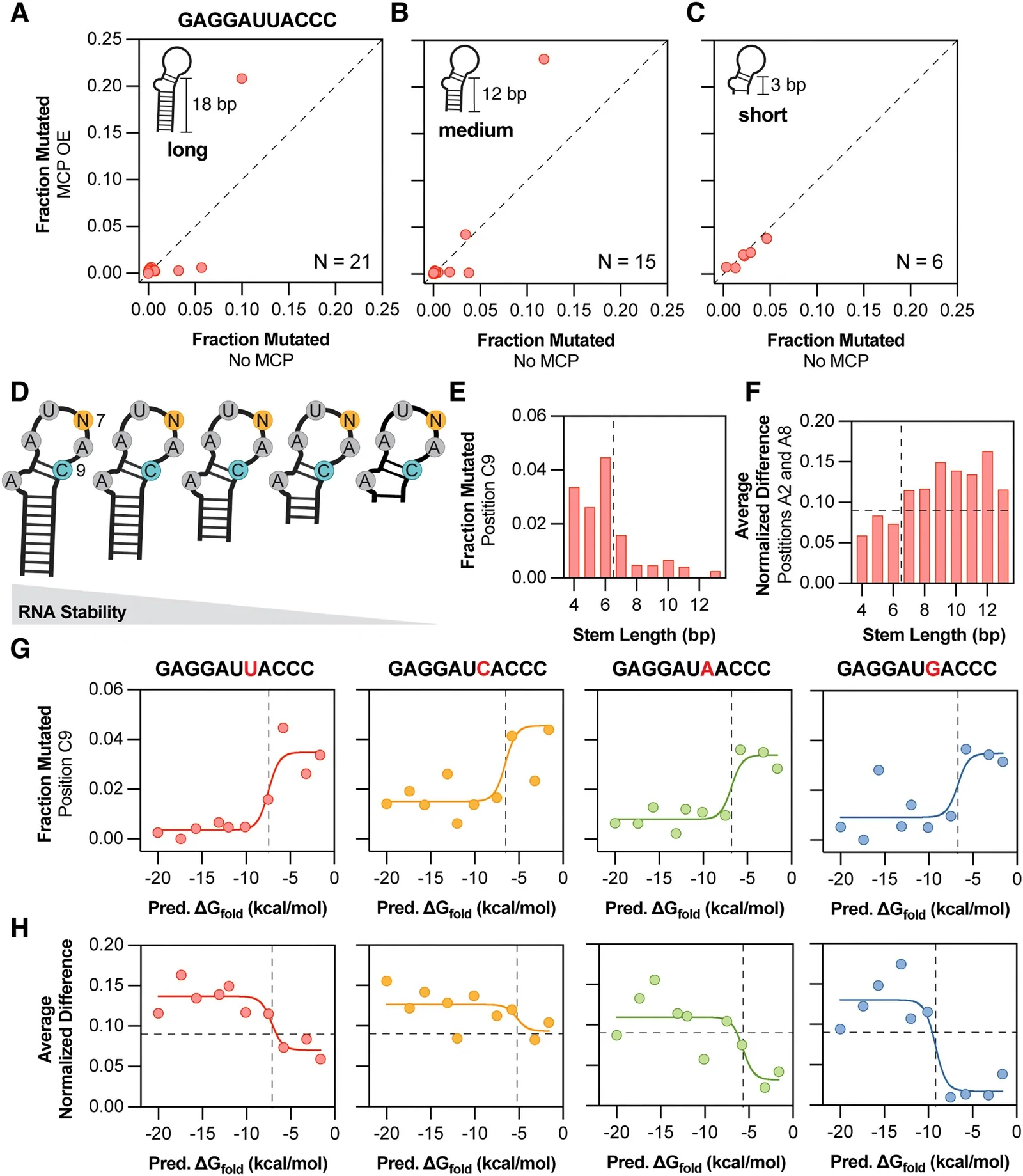
Stable RNA structures are required for MCP binding. Comparison of DMS-MaPseq reactivity (fraction mutated) at the same positions in the absence (*x*-axis) and presence (*y*-axis) of MCP, for the wild-type-like sequence GAGGAUUACCC with a long (**A**), medium (**B**), or short (**C**) stem with *75%* GC content. Each red circle represents one A or C base (*N*) averaged over two independent replicates. (**D**) A sublibrary of stem–loops with successively increasing stem length, from 4 to 13 bp, was designed. The position 7 was also varied to each of A, G, C, or U. (**E**) DMS-MaPseq reactivity of a base-paired position, C9 (red bars), for a set of wild-type loop sequences (GAGGAUUACCC) with successively longer stems. The vertical dashed lined represents the transition from unfolded hairpins (fraction mutated >0.02) to folded hairpins (fraction mutated <0.02). (**F**) MCP binding for the same set of sequences in panel (E), as measured by the average normalized difference to the no-MCP condition at protein-contacting positions 2 and 8 (red bars). The vertical dashed lined represented the transition from unfolded to folded hairpins from panel (E) and the horizontal dashed line represents statistically significant normalized difference (0.09) as established in Supplementary Fig. S1E. (**G**) Predicted folding free energies, as calculated by Mfold, versus measured RNA folding by DMS-MaPseq for each of the different tested tetraloop sequences and their varied stem lengths. Left to right, loop sequences with each of U, A, C, and G. Each colored circle represents the fraction mutated of C9 from a sequence of different stem length. The solid line represents the fit to nonlinear regression model relating *P*(unfolded) to ∆*G*_fold_ (see the “Materials and methods” section). The dashed vertical line represents the transition from folded to unfolded as calculated by non-linear regression. (**H**) Predicted folding free energy as in panel (G) versus MCP binding as measured by DMS-MaPseq. Left to right, same sequences as in panel (G). Each colored circle represents the average normalized difference of positions A2, A7/C7 (if applicable), and A8. The solid line represents the fit to nonlinear regression model relating *P*(bound) to ∆*G*_fold_ (see the “Materials and methods” section). The dashed vertical line represents the transition from bound to unbounded as calculated by non-linear regression and the horizontal dashed line represents statistically significant normalized difference (0.09) as established in Supplementary Fig. S1E.

The distribution of normalized difference values at A and C nucleotides located at positions directly surrounding and including the MS2 stem–loop between MCP overexpression replicates is centered close to 0 with a 95th percentile value of 0.09 (Supplementary Fig. S1D), indicating high reproducibility of the DMS signal across MCP-overexpressing samples. As MCP binding is expected to affect only a small number of MCP-contacting nucleotides rather than globally alter the stem–loop structure, the distribution of normalized difference values between samples with and without MCP overexpression is expected to largely overlap with the replicate distribution. The ±MCP comparison shows a similar bulk distribution but is skewed farther to the right with a 95th percentile value of 0.09, indicating that most structure is maintained through MCP overexpression but that MCP binding blocks DMS from reacting with a small number of key bases, increasing the difference between samples (Supplementary Fig. S1E).

After confirming that DMS modification was reduced at MCP-contacting positions upon the addition of MCP to the RNA library, the mutational profiles were examined for a library member that we hypothesized should bind MCP strongly in cells. The positional mutation fractions for a wild-type MS2 sequence (GAGGAUCACCC, long stem, medium GC) showed the least reactivity (lowest fraction mutated) at bases in the stem and the most reactivity (highest fraction mutated) at positions 5, 7, and 8, or the first, third, and fourth positions of the unpaired tetraloop, which are A, C, and A, respectively, as expected for unpaired positions (Fig. 1D). This difference in reactivity is consistent with a folded RNA hairpin. The accessibility of the open positions was significantly decreased upon overexpression of MCP (Fig. 1E) to the level observed in a no DMS control (Supplementary Fig. S1H). Concordantly, comparing the fraction of reads mutated at each position in the no-MCP condition versus the MCP overexpression condition shows a set of positions that fall below the *y* = *x* line (Fig. 1F), meaning that those positions were more accessible for modification before the addition of protein and are blocked by MCP binding upon its overexpression. The difference in reactivity is consistent with published structures of the MS2–MCP complex [44] and AlphaFold [45] models of our library sequences (Supplementary Fig. S3), which were used because the crystallized RNA differs in sequence from our hairpins. A8 and A2 make hydrogen bonding interactions with Ser47 and Thr45 on the protein on the Watson–Crick–Franklin face, hindering DMS reactivity at these positions. C7 makes π–π stacking interactions with Tyr85 where the Watson–Crick–Franklin face of the base faces the protein interface, blocking it from reacting with DMS. Overall, the differential reactivity at select RNA positions that make direct contact with protein residues indicates that DMS signal can be used to approximate MCP binding.

In contrast, performing the same analyses for a mutant MS2 sequence (GAGCAUGGGCC), predicted not to bind MCP, showed a relative lack of disruption to RNA structure at measurable positions (As or Cs), with higher accessibility at the unpaired position 2 (bulge) and 5 (first position in the tetraloop) (Fig. 1G). However, the mutation fraction of these positions is effectively unchanged upon the addition of MCP (Fig. 1H), with most positions closely following the *y* = *x* line when plotting the no-MCP and MCP overexpression samples against each other (Fig. 1I). This indicates that, as previously shown, MCP binding is sensitive to MS2 stem–loop mutation and demonstrates that DMS can simultaneously probe RNA folding and RBP binding preferences.

### Stable RNA structures are required for MCP binding

The parameters of the MS2 stem sequence were systematically varied to determine how stem length, GC content, and overall RNA folding contribute to MCP binding of MS2 stem–loops in cells. Changing the stem length (i.e. RNA stability, *K*_fold_) surrounding a wild-type-like MS2 sequence (GAGGAUUACCC) confirmed that a 3 base-pair stem is insufficient for MCP binding, while both 12 and 18 base-pair stems permit hairpin formation and MCP recognition (Fig. 2A–C). A long (18 base-pair) stem surrounding the wild-type-like AUUA tetraloop produced lower overall mutation fractions for bases in the stem and the first loop position, with increased reactivity at only two positions (A2 and A8), consistent with a folded hairpin structure inaccessible to DMS mutation. The mutation fractions of A2 and A8 both decrease completely to the level of sequencing noise upon MCP addition (Fig. 2A), consistent with MCP binding to this RNA structure. A medium (12 base-pair) stem shows a similar profile, with several key positions that become protected upon MCP overexpression (Fig. 2B). In contrast, the shortest stem (3 base-pairs) fails to fold sufficiently as indicated by several unpaired bases having a mutation fraction of 0.02 or greater. Consequently, the short stem does not bind MCP, showing a near-perfect correlation between the no-MCP and MCP-overexpression conditions (Fig. 2C). The GC content of the stem had little effect on MCP binding preferences for the same wild-type stem–loop (Supplementary Fig. S4A–C), and the stem-length dependencies were consistent across all wild-type or wild-type-like sequences in the library (Supplementary Fig. S4D). Mutant MS2 sequences showed smaller changes in response to MCP binding, with largest effects still observed for the medium and long stem lengths (Supplementary Fig. S4E).

To further probe how MCP binding depends on MS2 stem length, a sublibrary was constructed containing all base substitutions at position 7 within an otherwise wild-type stem–loop (GAGGAU_ACCC). For each loop, 10 stem mutants were generated by extending the stem 1–10 base pairs, for a total of 40 variants (Fig. 2D). Measuring the DMS mutation fraction in the absence of MCP at the first C past the loop (position C9), which should be base-paired and protected when the RNA folds correctly into a hairpin, revealed that increasing stem length progressively stabilizes RNA folding for a wild-type stem–loop sequence GAGGAUUACCC (Fig. 2E). The longest stems showed the least accessibility, or the most base pairing, with a stem length of around 7–8 base pairs required for the stem to fully form (Fig. 2E). MCP binding was quantified by averaging normalized differences between the ±MCP conditions at the three loop positions most directly involved in binding [Equation (1) and Supplementary Fig. S4F] and defined a binding threshold based on the 95th percentile of these values across the dataset (Supplementary Fig. S1E). Both of these metrics suggest that the MCP binding preferences closely follow the stem folding profile, with four additional stem base pairs required for detectable MCP binding to a wild-type stem–loop (Fig. 2F).

These analyses were expanded by using the mFold RNA prediction software [46] to calculate the predicted folding free energy (∆*G*_fold_) for each of the 4 loop sequences across their 10 stem lengths. DMS mutation fractions in the absence of MCP at position C9 scaled logarithmically with predicted ∆*G*_fold_ (Fig. 2G): below ~−7 kcal/mol, DMS reactivity at position C9 was nearly zero, indicating complete stem pairing. Above this value, DMS accessibility sharply increased before reaching a plateau, consistent with unfolded or unstable RNA stems (Fig. 2G). It is likely that the RNA hairpin structures are largely destabilized in cells relative to *in vitro* computational predictions [22, 47, 48], which contributes to the hypersensitive relationship between predicted structure and measured DMS reactivity. This relationship between DMS reactivity and folding stability is consistent with recent *in vitro* quantitative chemical probing studies showing that DMS measurements can report on the stability of tertiary contacts and provide estimates of RNA folding energetics [49, 50]. Comparing predicted ∆*G*_fold_ to average normalized differences (MCP binding) across the four loop families revealed the same trend, with MCP binding to each loop occurring only when ∆*G*_fold_ for the sequence is predicted to be below −7 kcal/mol (Fig. 2H). For the wild-type sequence GAGGAUUACCC, MCP binding decreased sharply at the same stem length where folding was lost (Fig. 2G and H; red), indicating a direct link between RNA structure and protein recognition. The other wild-type-like sequence GAGGAUCACCC showed similar RNA folding behavior, maintained binding across all stem lengths (Fig. 2G and H; orange), suggesting that the AUCA loop sequence has higher intrinsic affinity for MCP and requires less structural stabilization for recognition. The AUGA loop sequences (GAGGAUGACCC) required significantly more stable RNA structures, with ∆*G*_fold_ <−9 kcal/mol, before MCP binding was detected, although both sequences folded at the same −7 kcal/mol as the other two variants (Fig. 2G and H; blue). This indicates a slight disfavoring of MCP recognition of the non-wild-type AUGA loop sequences such that a more stable RNA structure is required for MCP to bind. Together these data indicate that folding itself is largely sequence-independent, whereas the stability at which MCP recognition is detected varies by loop sequence. Folding is therefore a prerequisite for recognition rather than a sufficient condition for it.

### Loop sequence is the second-biggest determinant of MCP binding preferences

After determining the importance of stable RNA structures for strong MCP–MS2 recognition, we examined the contribution of individual nucleotides to binding specificity. A wild-type-like MS2 sequence (GAGGAUUACCC, long stem, medium GC) was compared to all possible single-nucleotide substitutions at positions 2, 5, 7, and 8 (Fig. 3A). This systematic set of variants allows us to link single-base changes to their effects on MCP binding; for example, how mutating position 2 from A to U, C, or G alters binding.

**Figure 3.**
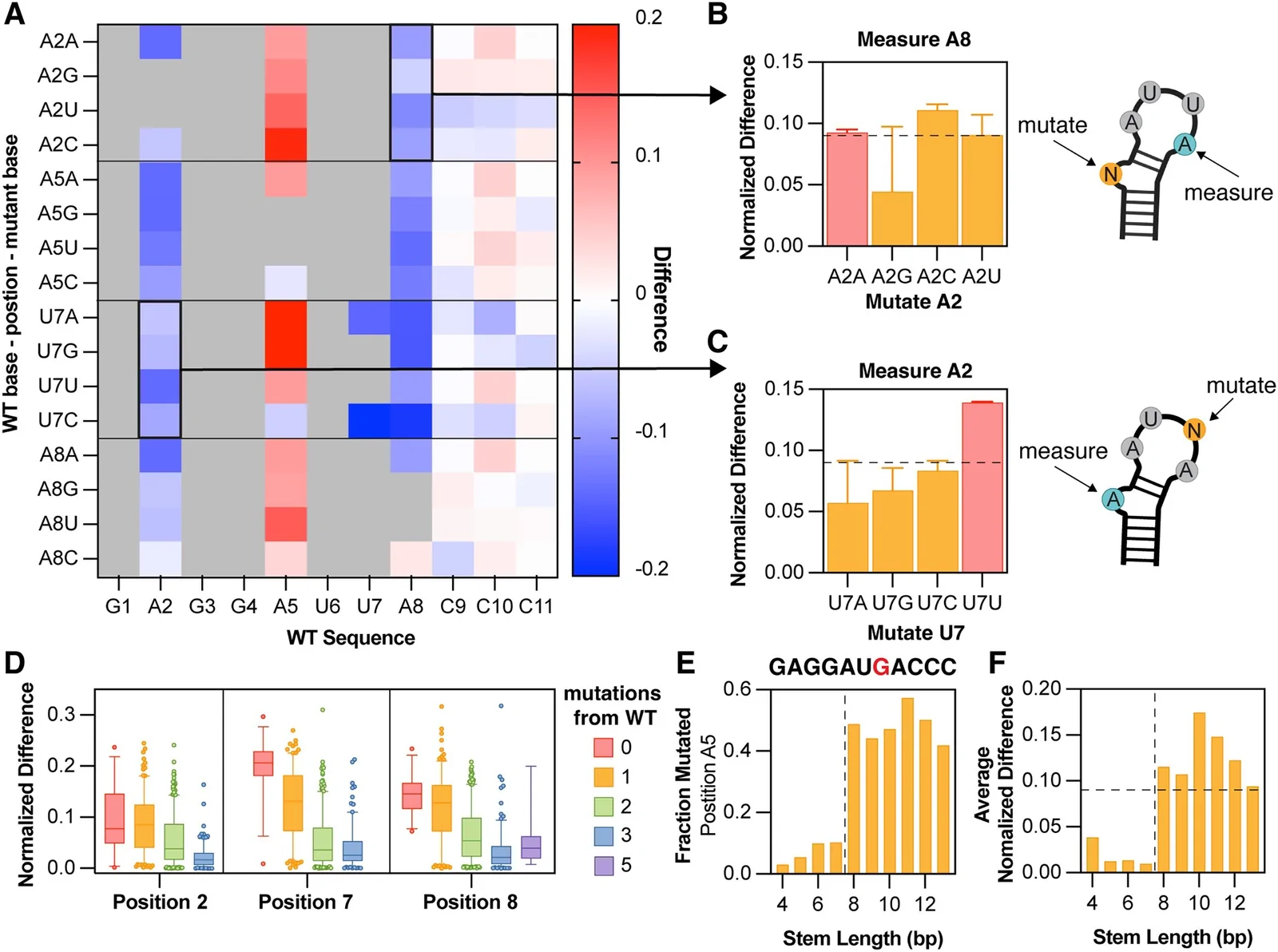
Loop sequence is the second-biggest determinant of MCP binding preferences. (**A**) Heatmap of the normalized difference in DMS-MaPseq signal from MCP overexpression to MCP absence at each stem–loop position (*x*-axis) in response to each mutation elsewhere in the sequence (*y*-axis). Gray boxes, non-measured positions (Gs or Us) or mutations that were not tested. (**B**) Quantification of the normalized difference in DMS-MaPseq signal upon MCP overexpression at position A8 in response to different base mutations made at the position 2. Each colored bar was averaged over two independent replicates. Error bars indicate the range. (**C**) Quantification of the normalized difference in DMS-MaPseq signal upon MCP overexpression at position A2 in response to different base mutations made at position 7. Each colored bar was averaged over two independent replicates. Error bars indicate the range. (**D**) Normalized difference in DMS-MaPseq signal at positions 2, 7, and 8. Boxes show first to third quartiles, whiskers show down to the 5th and up to the 95th percentile, and outliers are shown as circles. Colors indicate the number of mutations from the wild-type sequence (zero = red; one = orange; two = green; three = blue; and five = purple). (**E**) DMS-MaPseq reactivity (orange bars) of position A5 for a set of loop sequences (GAGGAUGACCC) with successively longer stems. The vertical dashed lined represents the transition from unbound hairpins to bound hairpins as calculated in Fig. 2G. (**F**) Average normalized difference to no MCP for positions A2 and A8 for the same set of sequences in panel (E). The vertical dashed lined represented the transition from unbound to bound hairpins from panel (E) and the horizontal dashed line represents statistically significant normalized difference (0.09) as established in Supplementary Fig. S1E.

Because MS2 sequences mutated at the bulge nucleotide (position 2) are known to be permissive to MCP binding, variation at this position provides a useful test case for our approach. Although DMS reactivity cannot be measured directly for G or U at this position (since they lack DMS-reactive sites on the Watson–Crick–Franklin face), other protein-contacting bases in the sequence can serve as proxies for how binding specificity is altered upon mutation. Position A8, which directly contacts the MCP dimer, was used as a constant “watch” position. Normalized differences at A8 showed that all bases except G are tolerated at position 2 (Fig. 3B), with G being heavily disfavored for binding. This is likely because a G bulge can form unintended base pairs with opposing stem Cs, disrupting proper MS2 folding and occluding MCP access to the exposed bulged nucleotide.

A similar strategy was applied to position 7, using A2 as the “watch” position. Here, the wild-type-like C is strongly preferred at position 7 (Fig. 3C), with all other bases being slightly disfavored. In contrast, using the A5 as the “watch” position shows the opposite trend, with large increases in accessibility upon MCP binding reflected by positive difference values in response to mutations at either position 2 or 7 (Fig. 3A). This suggests a conformational change in the MS2 loop upon MCP binding, potentially involving increased accessibility of the Watson–Crick–Franklin face or increased nucleophilicity of the N1 position of adenosine in the bound state (Supplementary Fig. S5), which can serve as an additional proxy for MCP binding. Notably, the largest increases in reactivity at A5 occur in response to mutations that substitute purines for pyrimidines or vice versa (Fig. 3A), suggesting that base identity influences both the conformation of the tetraloop in the bound state and its reactivity to DMS.

Together, these measurements demonstrate that comparing position-specific differences in DMS accessibility with and without MCP enables quantitative measure of RNA–protein interaction specificity. Among all variables tested, sequence similarity to the wild-type MS2 loop is the strongest determinant of MCP binding at the three positions that directly contact the protein: even a single mutation markedly reduces the normalized difference between the ±MCP conditions (Fig. 3D and Supplementary Fig. S5A). Sequences with two or more mutations away from wild type show little detectable binding, underscoring the high specificity of both the MS2 hairpin structure and its interaction with MCP (Fig. 3D).

In contrast, stem length and stem GC content have much smaller effects on binding. Increasing stem length beyond 12 base pairs does not significantly enhance binding at any of the three positions (Supplementary Fig. S5B–D, pink and orange boxes), although constructs with very short stems (3 bp; Supplementary Fig. S5B–D, green boxes) fail to bind MCP efficiently, consistent with the results in Fig. 2. Similarly, variation in stem GC content does not further affect binding beyond the effects caused by altering the loop sequence (Supplementary Fig. S5E–G). Finally, normalized difference values at other stem–loop positions reported not to directly interact with MCP remain largely unchanged across increasing mutational distances from wild type (Supplementary Fig. S5H), confirming that positions 2, 7, and 8 are the primary reporters of MCP binding.

Lastly, the increased accessibility at the first position of the tetraloop (A5) observed for pyrimidine to purine substitutions in Fig. 3A was examined in more detail. Using the stem–loop sequence GAGGAUGACCC, the DMS mutation fraction was measured directly in the presence of MCP, rather than the normalized difference to no MCP, and found a pronounced increase in A5 reactivity upon the addition of MCP (Fig. 3E) that coincides with the onset of MCP binding to an 8 base-pair stem (Fig. 3F). This increase is not observed for the wild-type GAGGAUUACCC sequence, despite measurable binding at a similar stem length (Fig. 2F and Supplementary Fig. S5I). While the mechanism underlying this differential A5 reactivity remains unresolved, these observations demonstrate that DMS-MaPseq can detect localized changes in RNA accessibility associated with protein binding, including at positions that do not directly contact the protein.

### DMS-MaPseq can quantify RBP binding across a wide range of concentrations

A system in which protein levels could be controlled over a wide range was next examined to evaluate the sensitivity of DMS-MaPseq for measuring binding specificity. Measuring binding at lower protein concentrations is an important test of this approach because most endogenous RBPs exist at subsaturating concentrations with respect to their RNA targets [47, 51, 52]. The same MCP dimer construct was fused to an inducible DHFR degron, a constitutive protein degradation tag that can be stabilized by increasing concentrations of TMP[53, 54]. Increasing TMP from 0 to 10 µM monotonically raised MCP levels (Supplementary Fig. S6A), although these levels remained well below the degronless MCP. While some residual MCP may persist at 0 TMP due to DHFR leakiness, the large differences between TMP-controlled and overexpressed concentrations allow measurements over a wide range of protein concentrations, including in a significantly subsaturating regime.

We calculated the normalized difference between the DMS mutation fractions at each position between each TMP dose and the 0 TMP condition. For a wild-type sequence GAGGAUCACCC, normalized differences increased monotonically at positions 7 and 8 with increasing TMP, reflecting non-saturating binding as MCP concentration increased (Fig. 4A). In contrast, after mutation at those positions (GAGGAUAUCCC), very little response to TMP was measured, indicating an overall non-binding sequence (Fig. 4C). When only position 7 was mutated to G (GAGGAUGACCC), there was much less response to TMP increase except for at position 5 (Fig. 4B), consistent with the purine-driven accessibility increase seen in Fig. 3E. At subsaturating MCP concentrations, MCP appears sufficient to induce a concentration-dependent conformational change at position A5 of the tetraloop but does not make measurable contacts with the A2 or A8. This might suggest that the bound conformation is different from that of the wild-type or wild-type-like MS2 sequences.

**Figure 4.**
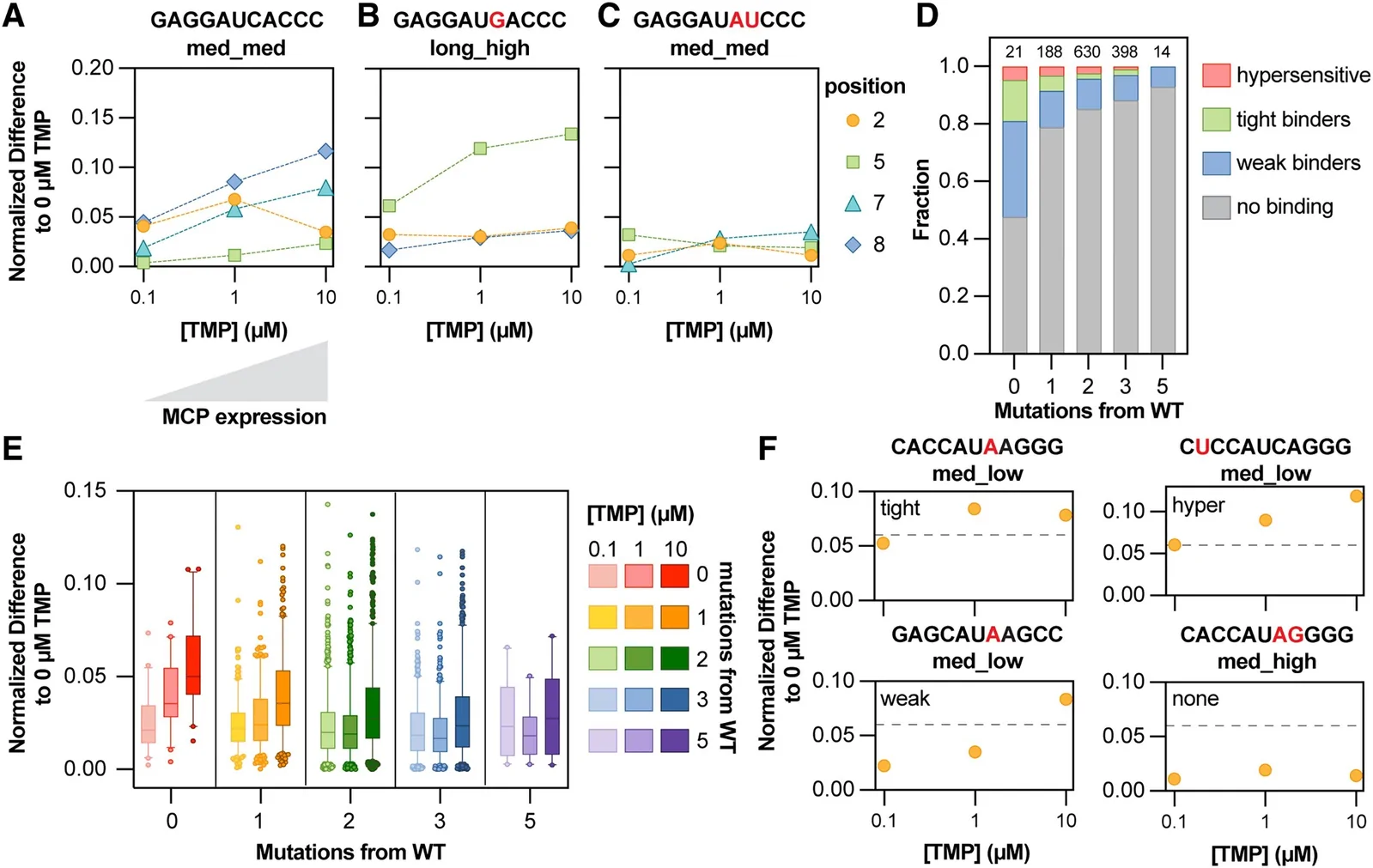
DMS-MaPseq can quantify RBP binding across a wide range of concentrations. TMP dose-response binding curves for (**A**) a wild-type MS2 sequence, (**B**) a sequence with one C to G mutation at position 7, and (**C**) a two-mutation, non-binding sequence. Each dashed line represents a different measured RNA nucleotide position that makes contact with MCP: position 2 (orange), position 5 (green), position 7 (turquoise), and position 8 (blue). *X*-axis, TMP dose; *y*-axis, normalized difference at the given position and TMP dose to the 0 TMP condition. The stem length and GC content of each construct is described under the sequence in the format {stem length}_{GC content}, e.g. medium length and medium GC for panel (A). (**D**) The fraction of sequences with increasing numbers of mutations away from wild-type MS2 classified as non-binding, weak, tight, or “hypersensitive.” The threshold for any binding activity between two given concentrations is set using the 95th percentile of the normalized differences between the 0.1 μM TMP replicates (0.06). (**E**) Average responsiveness to TMP for sequences with increasing number of mutations away from wild-type MS2, as measured by the average normalized difference to 0 TMP at the three positions (2, 7, 8). Boxes show first to third quartiles, whiskers show down to the 5th and up to the 95th percentile, and outliers are shown as circles. Only measurements with normalized difference <0.15 are shown. Colors indicate the number of mutations from the wild-type sequence (zero = red; one = orange; two = green; three = blue; and five = purple). Shading indicates the TMP dose (light = 0.1 μM; medium = 1 μM; and dark = 10 μM). (**F**) Dose responsiveness to TMP as measured by averaged normalized difference to 0 TMP for one in each binding category example sequences. Top left, classified as a “tight binder,” where MCP binding crosses the significance threshold (0.06) at 1 μM TMP and remains above 0.06 at higher concentrations. Top right, classified as “hypersensitive,” where MCP binding is above the significance threshold (0.06) at the lowest dose of TMP (0.1 μM) and remains above it at all doses. Bottom left, classified as a “weak binder,” where MCP binding does not cross the significance threshold (0.06) until 10 μM TMP, the highest concentration tested. Bottom right, non-binder, where MCP never crosses the significance threshold.

Overall, the average normalized difference for each sequence compared to the 0 TMP condition generally increased with TMP dose. Wild-type sequences and those with a single loop mutation showed the strongest response and highest overall binding (Fig. 4E and Supplementary Fig. S6C). Sequences with three or five mutations exhibited minimal binding but still showed modest TMP responsiveness, indicating that varying MCP concentration can reveal sub-threshold interactions that might otherwise be overlooked in bulk overexpression experiments.

To further quantify TMP responsiveness and binding dynamics across our library, we established an empirical significance threshold derived from biological replicates of the 0.1 µM TMP condition. The 95th percentile of normalized differences between replicates defines a strict signal-to-noise threshold of 0.06 (*P* < .05), below which reactivity changes represent measurement noise (Supplementary Fig. S6B). Using this threshold, we evaluated the responsiveness of each sequence across the titration relative to 0 µM TMP and classified the entire library into four distinct binding categories. Sequences exhibiting significant reactivity changes (≥0.06) already at 0.1 µM TMP were classified as hypersensitive (Fig. 4F, top right). Sequences that showed no significant binding (<0.06) at 0.1 µM TMP but crossed the threshold (≥0.06) at 1 µM TMP were designated as tight binders, saturating early, and reflecting a lower effective cellular *K*_d_ (Fig. 4F, top left). Sequences that remained within background noise (<0.06) through 1 µM TMP and only demonstrated significant binding (≥0.06) at 10 µM TMP were defined as weak binders (Fig. 4F, bottom left), while sequences that failed to cross the significance threshold across all tested concentrations were categorized as non-binders (Fig. 4F, bottom right). Notably, the weak, tight, and hypersensitive sequences shown in Fig. 4F were well above the binding threshold when incubated with degronless MCP (binding metrics of 0.2, top, and 0.16 bottom; threshold 0.09 (Fig. 2)). However, modulation of MCP concentration effectively revealed variabilities in their affinities in cells and demonstrated the need for a sensitive measurement technique to identify sub-saturating binding dynamics.

Mapping these classifications across mutational space revealed that the fraction of weak and tight binders each decreased with mutational distance, while the fraction of non-binders increased (Fig. 4D). Note that many sequences that are identical to WT in the loop portion (0 mutations from WT) contain short stems, and are expected not to bind. These classifications agreed with data using the degronless MCP, with tight sequences showing significantly higher normalized binding in the highly-expressed degronless MCP dataset (Supplementary Fig. S6D). Moreover, this trend remained when repeating the classification only on the sequences with medium and long stem lengths that are known to form appropriately folded hairpins in cells (Supplementary Fig. S6E). Together, these results show that tuning RBP abundance reveals affinity-dependent binding and conformational responses that are obscured under saturating conditions, underscoring the power of DMS-MaPseq to capture quantitative binding dynamics in cells.

### Quantitative framework for estimating fraction bound from MCP overexpression

Finally, we established a quantitative framework that converts the DMS-MaPseq signal for each RNA into an estimate of the fraction of RNAs bound by MCP inside cells. At each RNA position *j*, the measured mutation fraction reflects the sum of the probabilities (*P*) of unfolded, folded, and bound RNA states and the DMS reaction rate of that state [*k*_DMS_, Equation (2), the “Materials and methods” section]. The observed mutation fraction in each experiment for a given position is thus


(2) \begin{eqnarray*}
{\mathrm{MF}}_{j,\mathrm{observed}} &=& \big({P}_{\mathrm{unfolded}}{k}_{\mathrm{DMS},U,j}\left[ {\mathrm{ DMS}} \right] + {P}_{\mathrm{folded}}{k}_{\mathrm{ DMS},F,j}\left[ {\mathrm{ DMS}} \right] \\&&+ {{P}_{\mathrm{bound}}}{\mathrm{ }}{{k}_{\mathrm{DMS},B,j}}\left[ {\mathrm{ DMS}} \right] \big)t,
\end{eqnarray*}

where *k*_DMS, *U/F/B, j*_ is the rate of modification (per unit of DMS concentration, denoted by [DMS]) at position j when the RNA is in the unfolded, folded, and unbound, or bound states, respectively.

Using the stem length and folding analyses in Fig. 2, we have determined that every library member with a medium or long stem was determined to be folded in cells, evidenced by a lack of DMS reactivity at the paired stem bases. By restricting our analyses to only those library members, we know that the fraction of unfolded RNAs is negligible and approximate the fraction of unfolded RNAs to be close to 0 (*P*_unfolded _≈ 0). Additionally, only the RNA positions that directly contact MCP were used to approximate binding for this analysis, for which the rate of DMS reactivity is much lower when bound to MCP (*k*_DMS,*B*_≪*k*_DMS,*U*_). Under these constraints, the observed mutation fraction is proportional to the fraction of molecules that are folded but unbound at that position.

Thus, for each position *j*


(3) \begin{eqnarray*}
\mathrm{ M{{F}}_{\mathit{ j},\mathrm{observed}}} = {{P}_{\mathrm{folded}{\mathrm{ }}}}{{k}_{\mathrm{ DMS},F,j}}\left[ {\mathrm{ DMS}} \right]t.
\end{eqnarray*}

When the sequence is completely bound by MCP, *P*_bound_ = 1 and *P*_folded_ = 0; inversely, *P*_folded_ = 1 indicates a complete lack of binding at that position.

For each mutant sequence, a relative *P*_folded_ was computed by comparing its mutation fraction to the wild type at the same base. Importantly, this analysis was restricted to only common bases shared between the reference wild-type and each mutant sequence to ensure a constant rate of DMS modification, which is variable per base. All measured positions in the absence of MCP can be represented by *P*_folded_ = 1. This leads to the following equation for the differential probability of being folded (but not bound) between a mutant RNA compared to a WT sequence, based on the observed mutation fractions of each with and without MCP (see the “Materials and methods” section for full derivation):


(4) \begin{eqnarray*}
{\mathrm{\Delta }}{{P}_{\mathrm{folded}}} = \frac{{\mathrm{ MF}_{j,\mathrm{observed}}^{\mathrm{ mut}, + \mathrm{ MCP}}}}{{\mathrm{ MF}_{j,\mathrm{observed}}^{\mathrm{ mut}, - \mathrm{ MCP}}}} - \frac{{\mathrm{ MF}_{j,\mathrm{observed}}^{\mathrm{ WT}, + \mathrm{ MCP}}}}{{\mathrm{ MF}_{j,\mathrm{observed}}^{\mathrm{ WT}, - \mathrm{ MCP}}}}
\end{eqnarray*}

for each of the shared bases between the WT reference and each library member. The average was taken for each of those positions, which leaves us with a simplified *ΔP*_folded_, or fraction folded, for each sequence. Since these RNAs were assumed to be entirely folded, the fraction bound is simply


(5) \begin{eqnarray*}
{{f}_{\mathrm{bound}}} = 1{\mathrm{ }} - {\mathrm{ \Delta }}{{P}_{\mathrm{folded}}}.
\end{eqnarray*}

For this dataset, the wild-type reference sequenceGAGGAUCACCC, long stem, medium GC, shown in Fig. 1, was assumed to be completely bound: the DMS mutation fractions at positions 2, 7, and 8 decrease approximately to 0 upon the addition of MCP (Fig. 1D–F), enabling calculation of an absolute fraction bound. When applying this framework to wild-type and mutant RNA sequences for which the wild-type RNA is not fully bound, *f*_bound_ should instead be reported as *ΔP*_bound_, representing occupancy relative to the wild-type sequence.

This analysis provides a direct, quantitative measurement of the fraction of each RNA species that is bound in living cells. As expected, the fraction bound is very high (approaching 1) for each wild-type-like sequence in the library and decreases as the number of mutations away from the wild-type sequence increases (Fig. 5A). A total of 64 sequences were excluded because they yielded *f*_bound _values >1, an unphysical result that likely reflects measurement noise in low-signal DMS measurements rather than true occupancy. Additionally, the fraction bound is well-correlated with the qualitative normalized difference used in earlier analyses to rank binding strengths of different RNA sequences (Supplementary Fig. S7A). To further validate this occupancy metric, we compared the inferred fraction bound for library members that overlapped with a previous *in vitro* study of MS2–MCP binding [36]. Fraction bound in cells was strongly correlated with the corresponding *in vitro K*_d_ values (Fig. 5F), indicating that the occupancy measurements recapitulate established binding preferences across independent experimental contexts. Collapsing measurements across RNAs with the same stem–loop but different stem lengths or GC content reveals a clear mutational walk, as progressively diverging from the wild-type loop reduces the fraction bound in a near-monotonic fashion (Fig. 5B). These data allow an approximate percentage of how much each base contributes to overall binding. For example, a single mutation in the third tetraloop position from C to A (GAGGAUAACCC) decreases the fraction bound only by ∼5%–10% (Fig. 5B, third bar from left), but a single mutation in the fourth tetraloop position from A to C (GAGGAUCCCCC) decreases binding by ∼*20%* (Fig. 5B, fifth bar from left).

**Figure 5.**
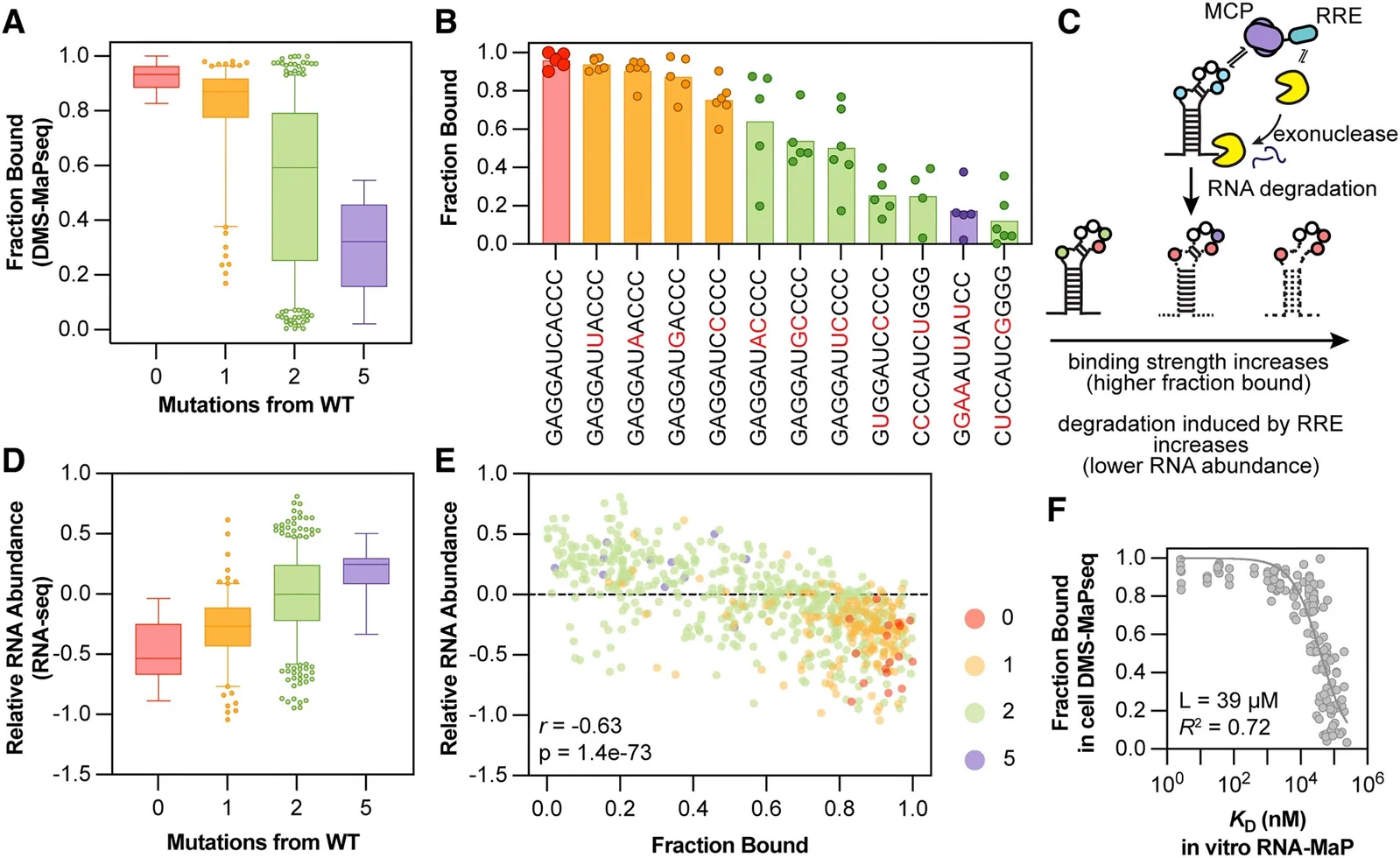
DMS-MaPseq predicts RBP occupancy and downstream gene regulation in living cells. (**A**) Measurements of the fraction of RNA molecules bound by MCP for library members with increasing numbers of mutations away from the MS2 consensus. Boxes show first to third quartiles, whiskers show down to the 5th and up to the 95th percentile, and outliers are shown as circles. Colors indicate the number of mutations from the wild-type sequence (zero = red; one = orange; two = green; and five = purple). Only measurements with fractions bound <1 are shown. (**B**) Measurements of fraction bound for example loop sequences. Circles show measurements for each loop with different stem lengths (medium, long) or GC contents (low, medium, high); bars show the average of those measurements. Bar colors show the number of mutations in each loop as compared to wild-type as in panel (A). (**C**) Schematic of RNA degradation experiment. An RNA-downregulatory effector domain (RRE) from the protein NANOS1 (amino acids 21–100) known to induce degradation of bound transcripts is fused to MCP, which is overexpressed in the presence of the same MS2 stem–loop library. As MCP occupancy increases, as measured by DMS-MaPseq, RNA abundance of a given library member should decrease as degradation is more effective. (**D**) Relative abundances of MS2 stem–loop library members, measured as a log_2_FoldChange between overexpression of the MCP–RRE construct compared to overexpression of RRE alone. Sequences are separated by increasing numbers of mutations away from the MS2 consensus as in panel (A). (**E**) Comparison between the predicted fraction bound of each stem–loop library member from DMS-MaPseq measurements and its measured relative abundance upon MCP–RRE overexpression. Colors represent mutational distance from the wild-type MS2, *n* = 667 sequences. Pearson *r* = −0.63; *P*-value = 1.4 × 10^−73^. (**F**) Comparison between previously measured *K*_D_ values extracted from *in vitro* binding experiments using MCP and MS2 variants (*x*-axis) and in-cell fraction bound measurements from this work for the same MS2 mutant sequences (*y*-axis). L represents the approximate concentration of MCP in cells and is extracted from the equation ${{f}_{\mathrm{bound}}} = \frac{L}{{L + {{K}_\mathrm{ D}}}}$, and *R*^2^ is the least-squares measurement of goodness of fit.

### Functional consequences of MCP specificity

To test whether the quantitative measurements of MCP–RNA binding could predict functional consequences inside cells, we fused MCP to a potent RNA-regulatory effector domain (RRE) from the protein NANOS1 known to trigger degradation of its bound RNA targets (Fig. 5C) [53]. The MCP–RRE fusion was overexpressed together with the same stem–loop library and quantified the steady-state relative abundance of each RNA variant by RNA-seq after 48 h compared to an MCP-alone control (Supplementary Fig. S7B). As expected, wild-type-like stem–loops, which were determined to be almost fully bound, showed the strongest depletion. RNA abundance increased progressively as the stem–loop accumulated more mutations (Fig. 5D), demonstrating that stem–loop sequence is a strong determinant of RNA fate.

The inferred fraction bound from DMS-MaPseq measurements quantitatively correlated with the degree of RNA depletion. Across all library members, an anticorrelation was observed between fraction bound and RNA abundance (*r* = –0.63; Fig. 5E). Generally, higher occupancy of the MCP–RRE corresponds to stronger RNA downregulation, albeit with considerable variability among individual library members, pointing at cellular phenomena that remain to be understood.

## Discussion

The development of this quantitative DMS-MaPseq framework provides an experimental platform for dissecting RNA–protein interactions at single-nucleotide resolution directly within living cells. By integrating RNA-centric metrics into RBP binding analyses, this system enables systematic probing of the functional consequences of cell state changes and mutational effects that alter both RNA structure and protein affinity in native contexts. This framework may facilitate the development of tunable tools or therapeutics whose RNA-binding properties can be predictably modulated by specific structural modifications or sequence changes.

Using the well-studied MCP–MS2 system as a test case, a library of systematically mutated MS2 stem–loops was generated to map how individual nucleotide identities and RNA structures contribute to MCP binding specificity. Although the relative contributions of RNA sequence and structure vary substantially across RBPs, the well-characterized MS2–MCP interaction provides an ideal benchmark for developing and validating quantitative approaches to infer protein occupancy from in-cell RNA structural measurements. These data show that single-nucleotide changes within the MS2 loop can substantially alter MCP binding, and that stem stability strongly influences the ability of the loop to adapt the correct conformation for recognition by MCP. To demonstrate this, we predicted minimum free-energy structures using mFold for each library member and found that nearly all variants with long and medium stem architectures were still predicted to adopt the canonical MS2 stem–loop despite their point mutations. In contrast, variants with the short stem architecture that never bind MCP rarely formed the canonical hairpin and instead frequently adopted alternative short stem–loops or other base-paired conformations, depending on the loop sequence and the availability of complementary nucleotides. The average ∆*G*_fold_ of the short, medium, and long variants are −2.53, −17.5, and −29.9 kcal/mol, respectively. Analogous to our successive stem shortening measurements in Fig. 2, these analyses demonstrate that stable canonical hairpin formation is imperative for MCP binding, and less stable RNA stems are more likely to form alternative structures that impede productive binding events. This coincidence is direct evidence that folding gates binding: the stem-stability threshold for MCP recognition is simply the stability at which the hairpin stops folding. Importantly, in the MS2–MCP system these data confirm that stable hairpin formation is required for productive protein binding, consistent with the established structural requirements of this interaction. These findings illustrate how RNA structure and sequence together determine recognition in this structured RNA–protein interaction. More broadly, these results demonstrate that incorporating experimentally measured RNA structural information can improve mechanistic understanding of RNA–protein interactions in systems where RNA conformation contributes to recognition [55, 56].

The TMP-inducible MCP system demonstrates that DMS-MaPseq is sensitive for binding detection across a wide range of varying protein concentrations, including low levels. This opens opportunities to investigate the interplay between protein concentration, binding affinity, and cellular dynamics. Shorter DMS treatments, or time-course experiments, could reveal transient or weaker interactions that might be missed under overexpression conditions, offering a method to probe the full spectrum of RNA–protein affinities. Although this study focuses on the well-characterized MS2–MCP interaction, the sensitivity of the approach suggests that it could be applied to additional RNA–protein systems. Whether occupancy can be robustly inferred for a given RNA–protein interaction will depend on the extent to which protein binding produces detectable changes in DMS accessibility, and will require empirical validation across diverse classes of RBPs. Applying the same DMS-MaPseq workflow *in vitro* and comparing it to in-cell measurements could benchmark predictions of RNA structure and RBP binding, account for cellular context effects, and refine models of RBP specificity. Such studies will also help define the classes of RNA–protein interactions for which DMS-derived occupancy measurements are most informative. This is particularly valuable for understanding interactions in crowded, structured, or highly dynamic RNA environments where *in vitro* affinities may not fully recapitulate behavior in cells [57]. DMS-MaPseq could also be combined with complementary approaches to measure RBP binding, such as UV crosslinking to provide orthogonal confirmation of binding events. While DMS reports on structural accessibility and indirect occupancy, UV crosslinking captures direct contact between RNA and protein [58], allowing for comparisons of transient and stable interactions. Integrating these methods could yield a more complete map of RBP–RNA interactions.

The quantitative framework produced here allows us to link the fraction of RNA bound by MCP directly to functional outcomes in cells. Using the MCP–RRE fusion experiments, hairpins with higher fractional occupancy experienced stronger RNA depletion, while those with reduced binding remained more abundant. This establishes that DMS-MaPseq-derived occupancy measurements are not merely descriptive, but predictive of regulatory output, allowing for a predictive functional model of RNA sequence and structural changes in cells. Importantly, modulation of intracellular MCP concentration with the TMP-inducible DHFR-MCP-RRE system demonstrated that the occupancy framework remains predictive across a broad range of protein concentrations, preserving the expected ordering of strong and weak binders and their corresponding RNA depletion across TMP doses (Supplementary Fig. S7C and D). Although occupancy explains a substantial fraction of the observed variation, additional factors likely contribute to the remaining differences in RNA abundance. In principle, this framework could be extended to additional RBPs and RNA effectors to test whether measured occupancy predicts downstream functional outcomes such as splicing efficiency, translation, or RNA decay. This approach also provides a new strategy for engineering tunable RNA tools: MS2 hairpins can now be designed to achieve graded MCP affinities by varying stem length, GC content, or specific loop nucleotides. Such synthetic RNA hairpins could then be used to fine-tune regulatory circuits, generate dose-dependent RNA–protein interactions, or develop RNA-based therapeutics whose function depends on predictable binding dynamics.

Several limitations of the present framework should be considered. The MS2–MCP interaction represents a favorable benchmark because its structural basis for recognition is well established, enabling direct validation of occupancy measurements against an independently characterized RNA–protein interaction. Chemical probing also has the potential to perturb the interactions it is intended to measure. While modification of the RBP can be a concern with chemical mapping methods, DMS was shown to not lead to significant changes, albeit assessed on different RBPs not MCP [43]. Additionally, our DMS concentration and time-course experiments showed no evidence that increasing modification perturbs MCP-dependent protection under the conditions used here. Although these observations support DMS as a minimally perturbative probe of RBP occupancy, this should be evaluated empirically when extending the approach to new RNA–protein sequences. More broadly, the ability to infer occupancy will depend on whether protein binding is sufficiently strong and whether it gives a reproducible change in DMS accessibility. RBPs that bind weakly, recognize predominantly single-stranded sequence motifs, or produce only subtle changes in local DMS accessibility may require greater sequencing depth, additional biological replicates, or alternative chemical probing reagents to accurately resolve occupancy. Although DMS-derived occupancy measurements provide a quantitative estimate of the fraction of RNA molecules bound in cells, absolute binding constants generally cannot be recovered without knowledge of the free intracellular protein concentration. Consequently, the current framework is best suited for measuring relative occupancy and effective binding preferences directly in living cells, while providing quantitative estimates of RNA folding equilibria under the conditions examined. Despite these limitations, this work establishes a quantitative framework for measuring RNA structure and protein occupancy directly in living cells, providing a foundation for future studies of RNA–protein interactions in their native cellular context.

## Supplementary Material

gkag1019_Supplemental_Files

## Data Availability

Raw and processed sequencing data have been deposited in the NCBI Gene Expression Omnibus and are accessible under accession number GSE344672. Processed sequence data are available in the Supplementary data. The MCP overexpression, DHFR-MCP, and DHFR-MCP-RRE plasmids are available on Addgene at IDs #242611, #242615, and #242616, respectively. All other requests for data and reagents will be fulfilled by the lead contact (ldhagler@tamu.edu) upon request.
