# Supplementary material for: Structural probing of RNA hairpins quantifies protein occupancy on RNA and links it to function in human cells
Source: Nucleic Acids Res. 2026 Oct 30;54(20):gkag1019. doi: 10.1093/nar/gkag1019 (PMC13720505; doi:10.1093/nar/gkag1019)
Supplement: Supplementary file 1 [file gkag1019_supplemental_files.zip › Thurm_NAR_SI_Revision_v2.pdf]

Supporting Information for:

Structural probing of RNA hairpins quantifies protein occupancy on RNA and links it to function in human cells

Abby R. Thurm[1], Tanya H. Nguyen[2], Jesus Damian Torres Campos[2], Matthew Mansfield[2], Lacramioara Bintu[3], and Lauren D. Hagler[2,*].

1 Biophysics Program, Stanford University School of Medicine, Stanford, CA 94305

2 Texas A&M University, Department of Chemistry, College Station, TX 77843

3 Department of Bioengineering, Stanford University, Stanford, CA 94305

*Corresponding author: ldhagler@tamu.edu

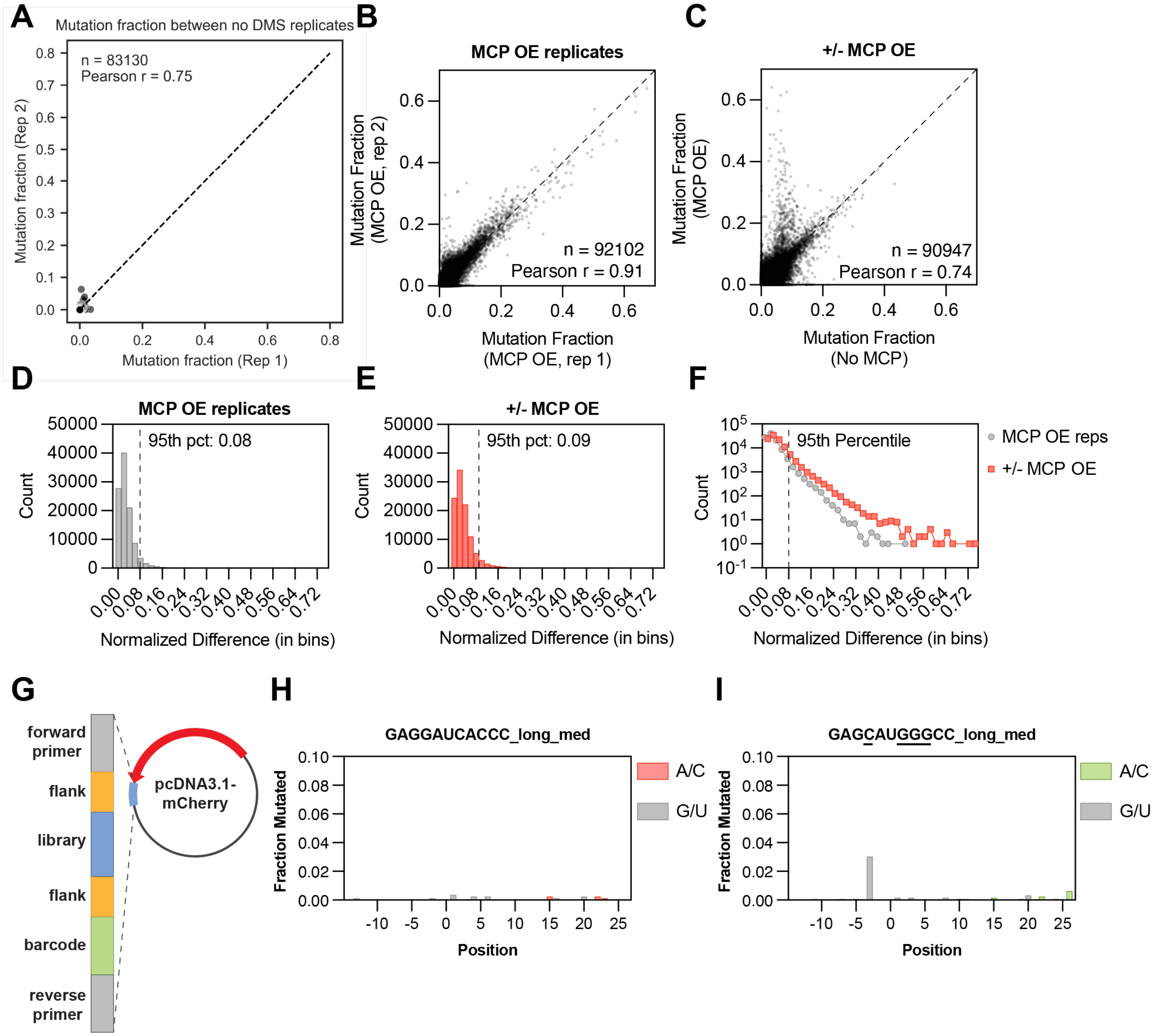

Figure S1. DMS-MaPseq replicates and metrics, related to Figure 1. (A) Mutation fractions as measured by DMS-MaPseq for all A and C bases in the MS2 library from samples transfected into HEK293T cells and not treated with DMS. X-axis, replicate 1; y-axis, replicate 2; N = 83,130 positions measured. (B) Mutation fractions as measured by DMS-MaPseq for all A and C bases in the MS2 library from samples transfected into HEK293T cells with overexpression of MCP, treated with DMS. X-axis, replicate 1; y-axis, replicate 2; N = 92,102 positions measured. (C) Comparison of mutation fractions, averaged over replicates, for A and C bases in the MS2 library from samples transfected into HEK293T cells with (y-axis) and without (x-axis) MCP overexpression, treated with DMS. N = 90,947 unique positions measured. (D) Distribution of normalized difference values between two replicate samples of A and C bases in the MS2 library transfected into HEK293T cells with overexpression of MCP, calculated using Eq. 1. X-axis, normalized difference value; vertical line, 95th percentile value and significance cutoff. (E) Distribution of normalized difference values between samples of A and C bases in the MS2 library transfected into HEK293T cells with and without overexpression of MCP, calculated using Eq. 1. X-axis, normalized difference value; vertical line, 95th percentile value and significance cutoff. (F) Combined normalized differences from (D) and (E) with the count on a log-scale. (G) Library design for the MS2 hairpin

library. (**H**) DMS reactivity (fraction mutated) at all positions of a mutated MS2 stem-loop **GAGGAUCACCC** in the presence of MCP with no DMS treatment. A and C bases are represented by red bars. G and U bases are represented by grey bars. (**I**) DMS reactivity (fraction mutated) at all positions of a mutated MS2 stem-loop **GAGCAU<u>GG</u>GCC** in the presence of MCP with no DMS treatment. A and C bases are represented by green bars. G and U bases are represented by grey bars.

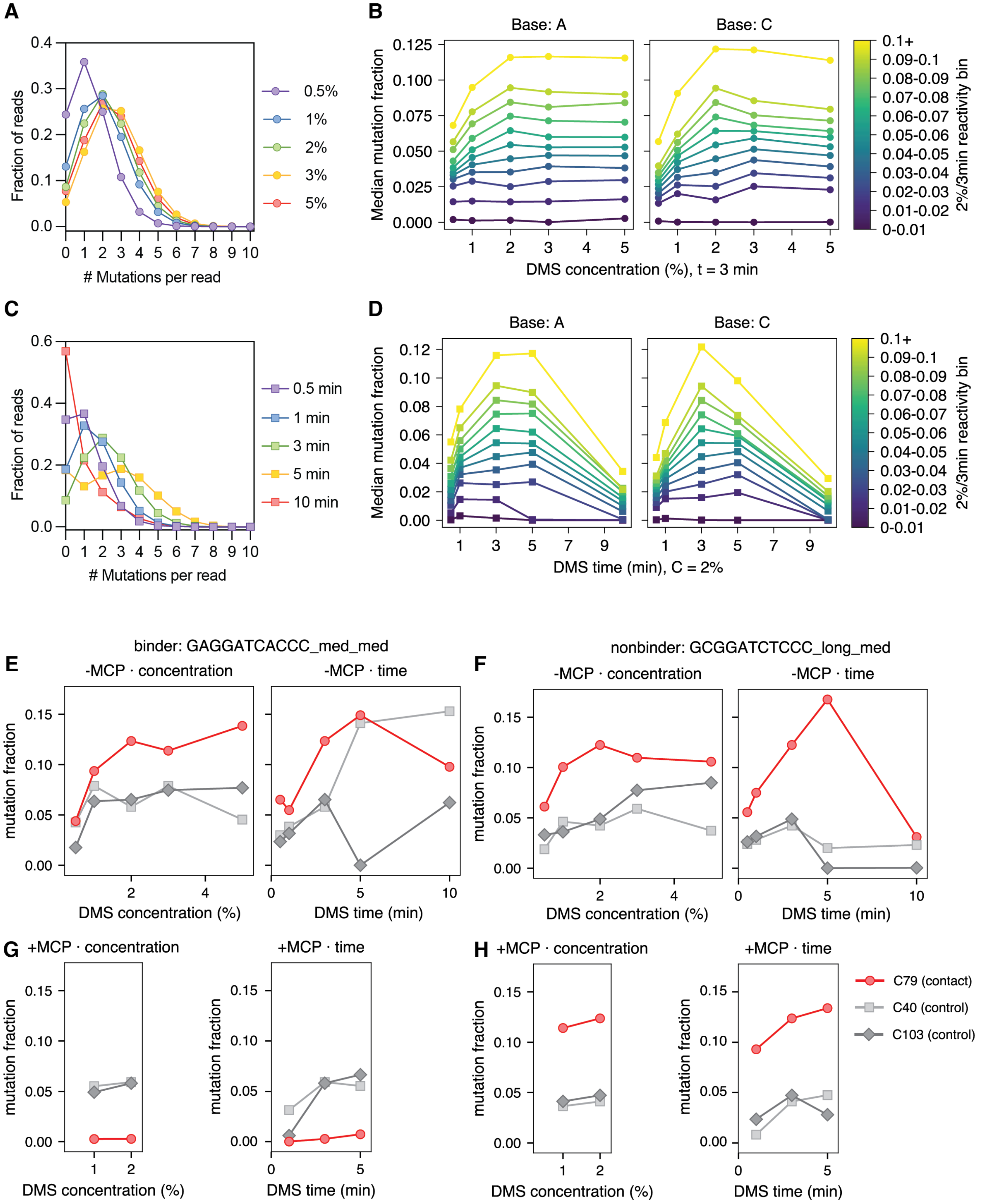
A
Fraction of reads
Mutations per read
0.5%
1%
2%
3%
5%
B
Base: A
Base: C
Median mutation fraction
DMS concentration (%), t = 3 min
2%/3min reactivity bin
0.1+
0.09-0.1
0.08-0.09
0.07-0.08
0.06-0.07
0.05-0.06
0.04-0.05
0.03-0.04
0.02-0.03
0.01-0.02
0-0.01
C
Fraction of reads
Mutations per read
0.5 min
1 min
3 min
5 min
10 min
D
Base: A
Base: C
Median mutation fraction
DMS time (min), C = 2%
2%/3min reactivity bin
E
binder: GAGGATCACCC_med_med
-MCP · concentration
-MCP · time
mutation fraction
DMS concentration (%)
DMS time (min)
F
nonbinder: GCGGATCTCCC_long_med
-MCP · concentration
-MCP · time
mutation fraction
DMS concentration (%)
DMS time (min)
G
+MCP · concentration
+MCP · time
mutation fraction
DMS concentration (%)
DMS time (min)
H
+MCP · concentration
+MCP · time
mutation fraction
DMS concentration (%)
DMS time (min)
C79 (contact)
C40 (control)
C103 (control)

Figure S2: In cell DMS-MaPseq control experiments. (A) Number of mutations per read at varying concentrations of DMS from 0.5-5% (v/v) DMS in DMEM. Reaction time was held constant at 3 min. The x-axis shows the number of mutations per read, and the y-axis is the fraction of reads for that concentration. (B) Median mutation fraction at varying DMS concentrations as in (A). A and C bases were binned using the working conditions (2% v/v, 3 min) and tracked across varying DMS concentrations, taking the median mutation fraction of each bin at each concentration. The solid lines are shown to connect bins, not to imply fit. (C) Number of mutations per read at reaction times 0.5-10 min. DMS concentrations was held constant at 2% (v/v) DMS in DMEM. The x-axis shows the number of mutations per read, and the y-axis is the fraction of reads for that concentration. (D) Median mutation fraction at varying reaction times as in (C). A and C bases were binned using the working conditions (2% v/v, 3 min) and tracked across reaction times, taking the median mutation fraction of each bin at each time point. (E,F) Changes in mutation fraction for three positions C79 (that contacts the protein) as well as C40 and C103 which do not contact the protein at varying DMS concentration and reaction time in the absence of MCP for a representative "binder" GAGGATCACCC_med_med (E) and "nonbinder" GCGGATCTCCC_long_med (F). (G) The same construct and positions from (E) in the presence of MCP at varying DMS concentration and reaction time. (H) The same constructure and position from (F) in the presence of MCP at varying DMS concentration and time. The solid lines throughout are used to connect points, not to imply fit.

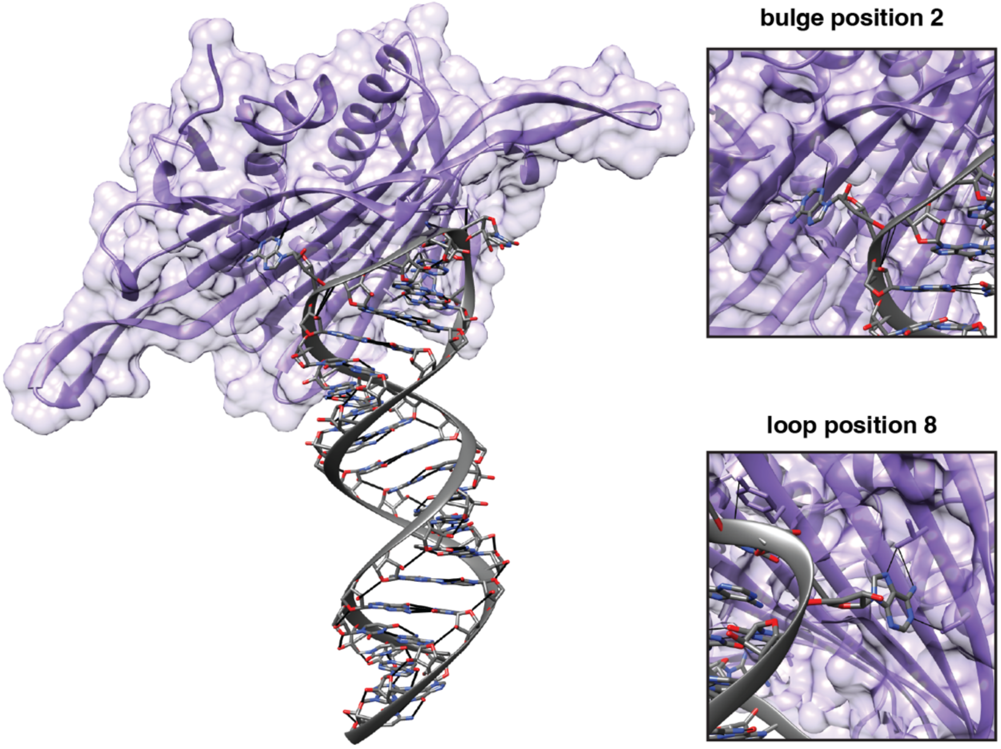

Figure S3. Representative AlphaFold model of an RNA-protein complex. The structure of GAGGAUCACCC_med_med was predicted with two copies of MCP (Uniprot: P03612) using the AlphaFold server.[1] The resulting complex was visualized and analyzed in Chimera.[2] (left) The protein, MCP, is shown in purple. The RNA bases are shown in grey. The black lines indicated predicted hydrogen bonds based on the angle and distance of the hydrogen bond donor and acceptor. (top right) Hydrogen bonding from threonine 45 to the N1 position of A2 and from serine 47 to the N3 position of A2. (bottom right) Hydrogen bonding from threonine 45 to the N6 and N7 positions of A8 and from serine 47 to the N1 position of A8.

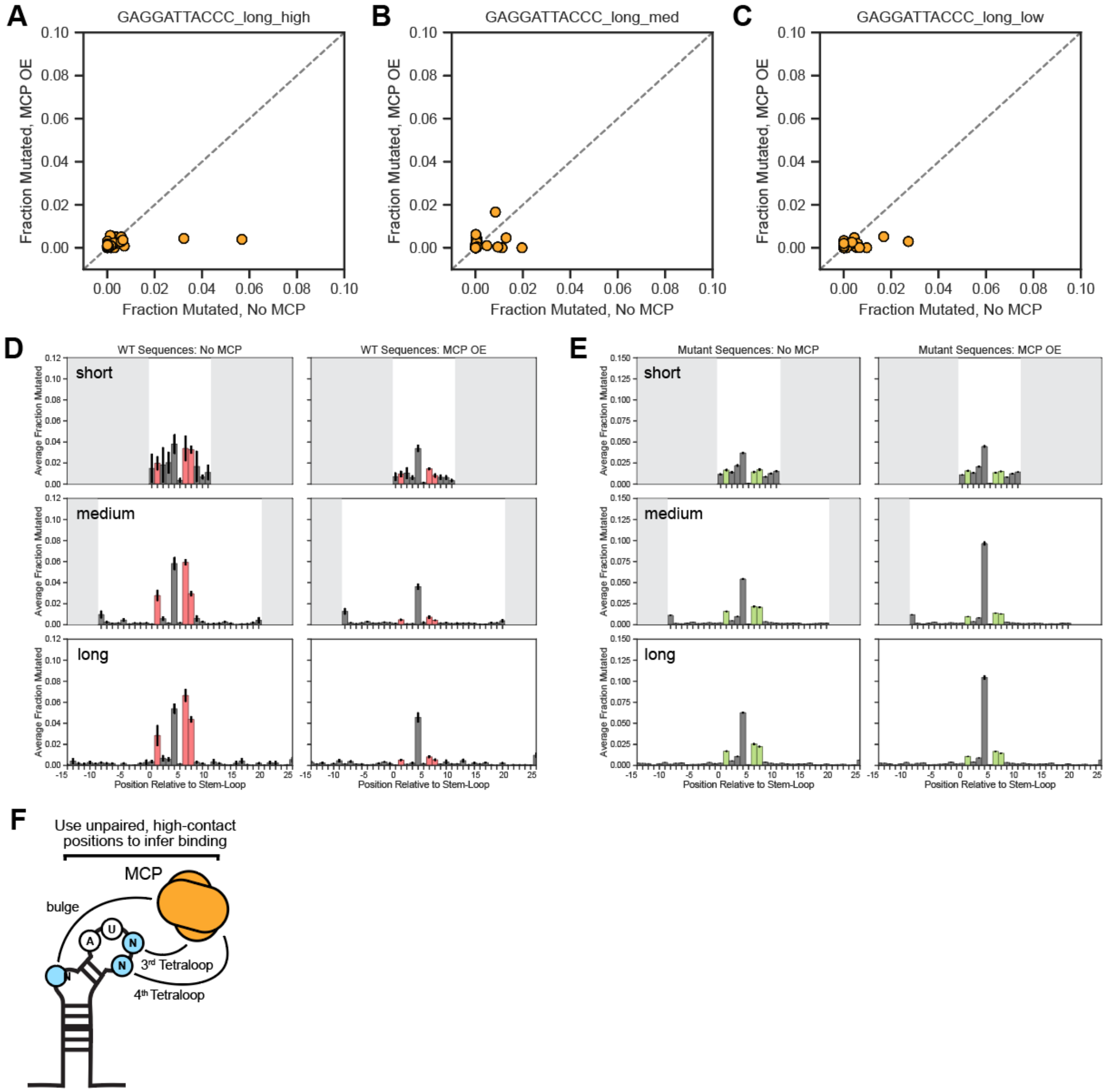

Figure S4. DMS-MaPseq measurements of library members with changing stem lengths and GC content, related to Figure 2. (A-C) Comparison of DMS-MaPseq signal at the same positions in the absence (x-axis) and presence (y-axis) of MCP, for the wild-type-like sequence GAGGAUUACCC with a long stem and high (A, 80%), medium (B, 50%), or low (C, 30%) GC content. (D) Accessibility at all positions of all wild-type MS2 library sequences with short (top row), medium (middle row), or long (bottom row) stems, in the absence (left) and presence (right) of MCP, as measured by the DMS-MaPseq mutation fraction at each position. (E) Accessibility at all positions of all mutant MS2 library sequences with short (top row), medium (middle row), or long (bottom row) stems, in the absence (left) and presence (right) of MCP, as measured by the DMS-MaPseq mutation fraction at each position. (F) Schematic of binding metric calculation. Normalized difference values at the three positions that directly contact MCP - the bulge, third tetraloop, and fourth tetraloop positions - are averaged for an "average normalized difference" and qualitative metric to compare binding strengths across library members.

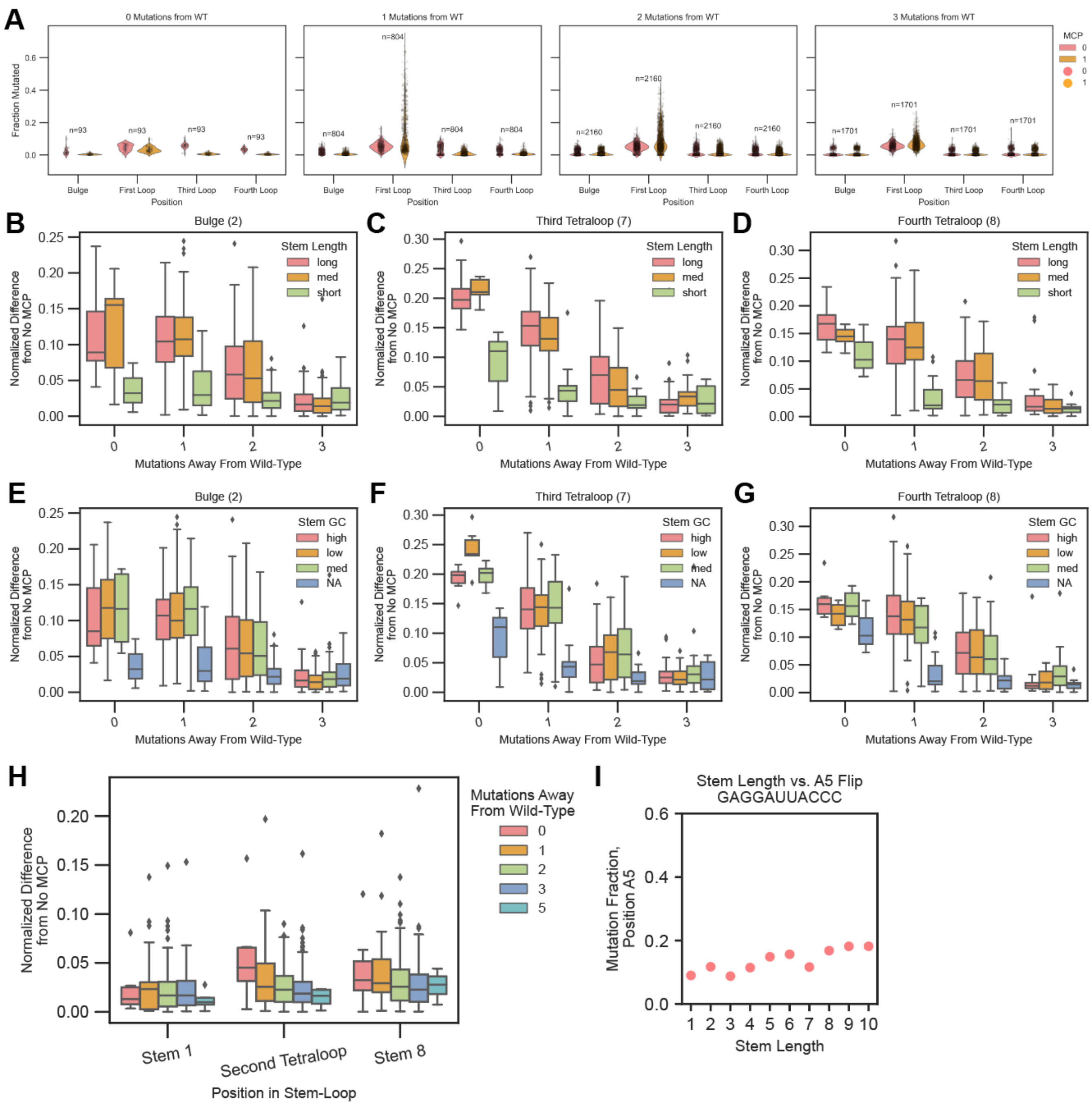

Figure S5. Mutational distance decreases binding affinity, related to Figure 3. (A) Fraction mutated values for samples without (pink) and with (orange) MCP, at the bulge position and the first, third, and fourth loop positions in the MS2 tetraloop. L-R, samples with 0, 1, 2, and 3 mutations away from the wild-type consensus. (B-D) Normalized difference in DMS-MaP signal at the bulge (B), third tetraloop (C), and fourth tetraloop (D) positions, separated by the number of mutations that differ from wild-type MS2, and colored by stem length (long: 15bp, medium: 9bp, short: 0bp). Boxes show first to third quartiles, whiskers show 1.5 x IQR, and outliers are shown as diamonds. (E-G) Normalized difference in DMS-MaP signal at the bulge (E), third tetraloop (F), and fourth tetraloop (G) positions, separated by the number of mutations that differ from wild-type MS2, and colored by stem GC content (high: 80%, medium: 50%, low: 30%, NA: 0bp stem). (H) Normalized difference in DMS-MaPseq signal at control positions: the first position in the stem, the second tetraloop position that contacts MCP less consistently, and the eighth stem position 3' to the loop. Sequences are separated and colored by the number of mutations in a sequence that differ from the wild-type MS2. (I) DMS-MaPseq measurements of first tetraloop position A5 for a set of wild-type loop sequences (GAGGAUuACCC) with successively longer stems. Y-axis, DMS-MaPseq mutation fraction at position A5; x-axis, stem length of stem-loop sequence.

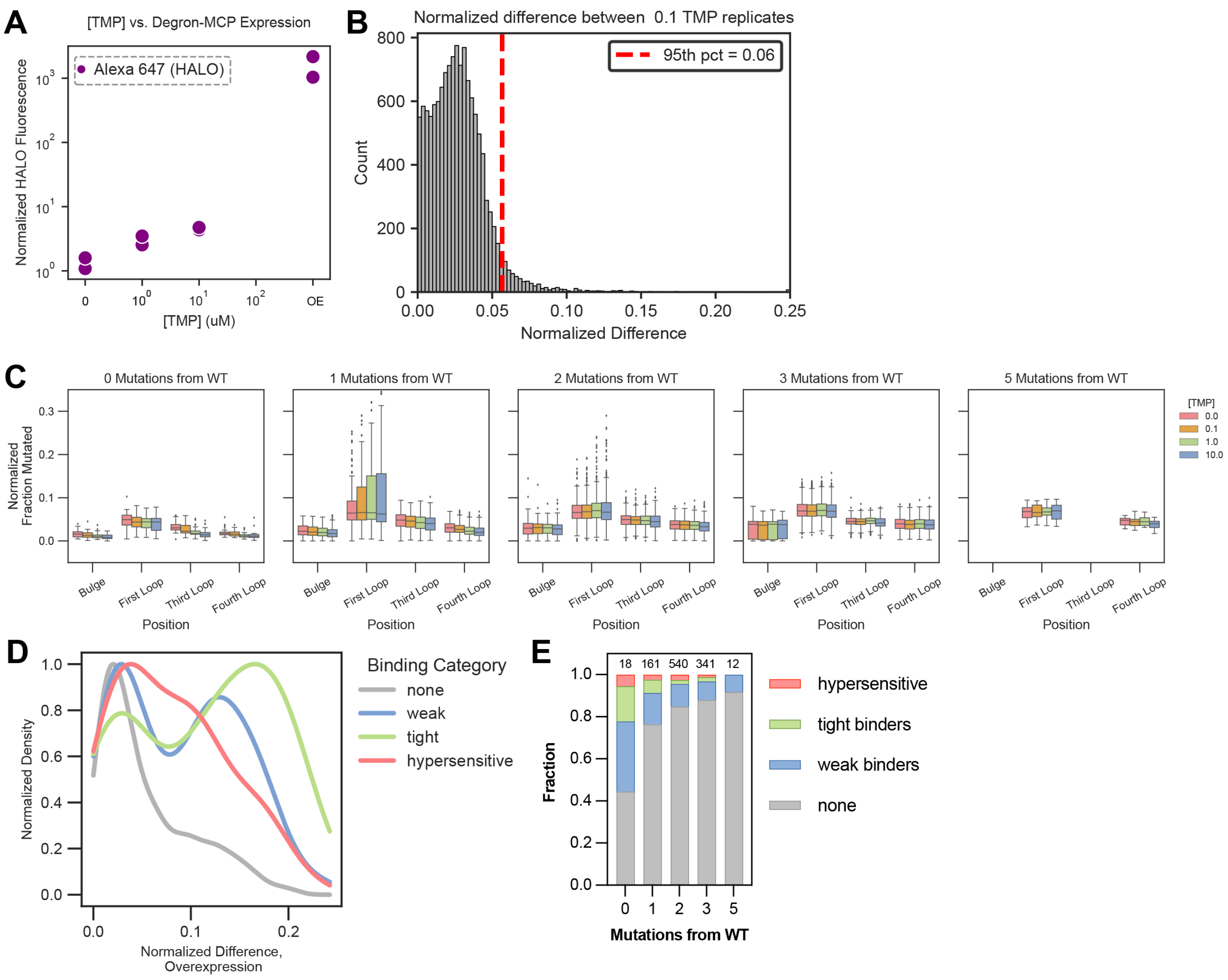

Figure S6. Details of DHFR-MCP fusion and TMP dosing with DMS-MaPseq experiments, related to Figure 4. (A) Concentration of DHFR-MCP fusion at different TMP doses, as measured by JaneliaTag-Alexa647 staining of the HALO ligand directly fused to MCP. X-axis, TMP concentration in μM; OE = overexpression of a HaloTag-MCP construct with no DHFR. Y-axis, HaloTag fluorescence normalized to a stained but non-expressing control. (B) Distribution of normalized difference values between two replicate samples of A and C bases in the MS2 library transfected into HEK293T cells with overexpression of DHFR-MCP in the presence of 0.1 μM TMP, calculated using Eq. 1. X-axis, normalized difference value; vertical line, 95th percentile value and significance cutoff. (C) Fraction mutated values normalized within each replicate for samples at the bulge position and the first, third, and fourth loop positions in the MS2 tetraloop at different TMP doses. Sequences are separated and colored by TMP dose (0, 0.1, 1, and 10 μM). L-R, samples with 0, 1, 2, 3, and 5 mutations away from the wild-type consensus. Boxes show first to third quartiles, whiskers show 1.5 x IQR, and outliers are shown as diamonds. (D) Distribution of average normalized difference (binding) values in the MCP overexpression experiments shown in Figure 1-3, colored by determined binding category using TMP dosing information as in Figure 4D-4G. (E) The fraction of sequences with medium or long stems (no short stems) with increasing numbers of mutations away from wild-type MS2 classified as non-binding, weak, tight, or 'hypersensitive', as in **Figure 4D**. The threshold for any binding activity between two given concentrations is set using the 95th percentile of the normalized differences between the 0.1 μM TMP replicates (0.06).

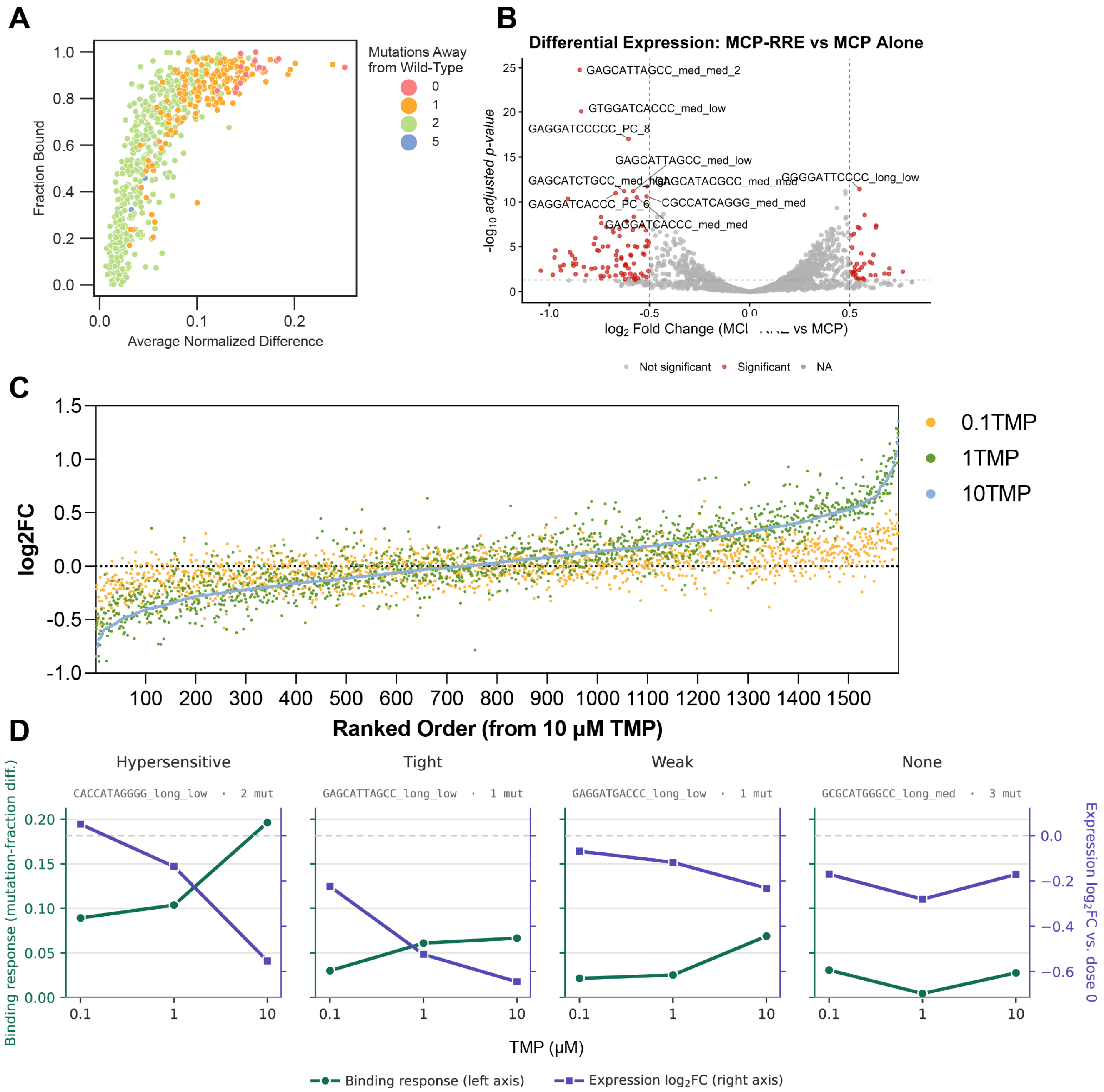
A
Fraction Bound
Average Normalized Difference
Mutations Away from Wild-Type
0
1
2
5
B
Differential Expression: MCP-RRE vs MCP Alone
-log10 adjusted p-value
GAGCATTAGCC_med_med_2
GTGGATCACCC_med_low
GAGGATCCCCC_PC_8
GAGCATTAGCC_med_low
GAGCATCTGCC_med_high
GAGCATACGCC_med_med
GGGGATTCCCC_long_low
GAGGATCACCC_PC_6
CGCCATCAGGG_med_med
GAGGATCACCC_med_med
Not significant
Significant
NA
C
log2FC
0.1TMP
1TMP
10TMP
Ranked Order (from 10 μM TMP)
D
Hypersensitive
Tight
Weak
None
CACCATAGGGG_long_low · 2 mut
GAGCATTAGCC_long_low · 1 mut
GAGGATGACCC_long_low · 1 mut
GCGCATGGGCC_long_med · 3 mut
Binding response (mutation-fraction diff.)
Expression log2FC vs. dose 0
TMP (μM)
Binding response (left axis)
Expression log2FC (right axis)

Figure S7. Transformation of DMS-MaPseq values to fraction occupancy predicts RNA degradation, related to Figure 5. (A) Comparison of average normalized difference values (x-axis) and transformed fraction bound values (y-axis) for all measurable sequences in the MS2 library (medium and long stem only, removing un-like bases). Sequences are colored by the number of mutations in a sequence that differ from the wild-type MS2. (B) Differential expression of all MS2 library members as measured by RNA-seq during overexpression with MCP alone or the degrading synthetic MCP-RRE fusion. X-axis, relative RNA abundance in log_2FoldChange; smaller values mean lower abundance in the MCP-RRE overexpression sample. Y-axis, -log_{10} adjusted p-value for each sequence. Significantly differentially expressed transcripts, with a cut off of +/- 0.5 log_2FoldChange and -log_{10} adjusted p-value of 1, are colored in red. (C) Change in measured MS2 library RNA levels in the presence of DHFR-MCP-RRE compared to the no TMP condition (y-axis) ranked by biggest change at the highest TMP dose (10 μM), colored by TMP dose (0.1, 1, 10 μM). (D) Selected example MS2 library sequences in the presence of DHFR-MCP-RRE belonging to the binding categories delineated in **Figure 4** (L-R: hypersensitive, tight, weak, non-binder). X-axis, TMP dose; green y-axis, normalized difference (binding metric); blue y-axis, relative library RNA levels compared to the no-TMP condition.

Table S1. Completed Library Sequences.

Table S2. Sample Conditions, related to Supplementary Dataset

Sample	MCP Condition	TMP conc.	% DMS (v/v)	Time (min)
**ART295**	MCP-DHFR	0 uM	2%	3
**ART296**	MCP-DHFR	0.1 uM	2%	3
**ART297**	MCP-DHFR	1 uM	2%	3
**ART298**	MCP-DHFR	10 uM	2%	3
**ART299**	MCP-DHFR	0 uM	2%	3
**ART300**	MCP-DHFR	0.1 uM	2%	3
**ART301**	MCP-DHFR	1 uM	2%	3
**ART302**	MCP-DHFR	10 uM	2%	3
**L304**	n/a	n/a	2%	3
**L305**	MCP	n/a	2%	3
**L306**	MCP	n/a	2%	3
**L311**	MCP	n/a	0%	0
**L312**	MCP	n/a	0%	0
**L313**	MCP-RRE	n/a	0%	0
**L314**	MCP-RRE	n/a	0%	0
**Lib30sec**	MCP	n/a	2%	0.5
**Lib1min**	MCP	n/a	2%	1
**Lib5min**	MCP	n/a	2%	5
**Lib10min**	MCP	n/a	2%	10
**Lib05per**	MCP	n/a	0.5%	3
**Lib1per**	MCP	n/a	1%	3
**Lib2per**	MCP	n/a	2%	3
**Lib3per**	MCP	n/a	3%	3
**Lib5per**	MCP	n/a	5%	3

**MCP1per3min**	MCP	n/a	1%	3
**MCP2per1min**	MCP	n/a	2%	1
**MCP2per3min**	MCP	n/a	2%	3
**MCP2per5min**	MCP	n/a	2%	5
**NANOS0TMP**	MCP-RRE-DHFR	0 uM	0%	0
**NANOS01TMP**	MCP-RRE-DHFR	0.1 uM	0%	0
**NANOS1TMP**	MCP-RRE-DHFR	1 uM	0%	0
**NANOS10TMP**	MCP-RRE-DHFR	10 uM	0%	0

Supplementary Note: High-Throughput RNA Library Design

We systematically designed the MS2-MCP library to probe how RNA sequence and structure influence RNA-RNA and RNA-protein interactions. The library samples a defined parameter space, enabling controlled, interpretable perturbations and parallel analysis of thousands of RNA variants under uniform experimental conditions.

To maximize accuracy, reproducibility, and interpretability, the library incorporates several design features: (1) **Accurate sequence identification** - Each RNA variant is linked to a unique 12-nt barcode with a minimum Hamming distance of three, enabling reliable discrimination among closely related sequences and minimizing misassignment from sequencing errors.

(2) **Minimized PCR bias** - All constructs share standardized flanking regions and primer-binding sites and are amplified using low-cycle PCR, ensuring read counts reflect biological signal rather than amplification efficiency.

(3) **High signal-to-noise** - Libraries are sequenced at high depth (typically ≥10,000 reads per variant), with measurement uncertainty estimated by bootstrapping sequencing reads.

(4) **Built-in replicability** – RNAs sequences are represented by multiple independent barcodes, providing internal technical replication, and biological replicates across independent experiments capture variability in cellular context.

REFERENCES:

(1) Abramson, J.; Adler, J.; Dunger, J.; Evans, R.; Green, T.; Pritzel, A.; Ronneberger, O.; Willmore, L.; Ballard, A. J.; Bambrick, J.; Bodenstein, S. W.; Evans, D. A.; Hung, C.-C.; O'Neill, M.; Reiman, D.; Tunyasuvunakool, K.; Wu, Z.; Žemgulytė, A.; Arvaniti, E.; Beattie, C.; Bertolli, O.; Bridgland, A.; Cherepanov, A.; Congreve, M.; Cowen-Rivers, A. I.; Cowie, A.; Figurnov, M.; Fuchs, F. B.; Gladman, H.; Jain, R.; Khan, Y. A.; Low, C. M. R.; Perlin, K.; Potapenko, A.; Savy, P.; Singh, S.; Stecula, A.; Thillaisundaram, A.; Tong, C.; Yakneen, S.; Zhong, E. D.; Zielinski, M.; Žídek, A.; Bapst, V.; Kohli, P.; Jaderberg, M.; Hassabis, D.; Jumper, J. M. Accurate Structure Prediction of Biomolecular Interactions with AlphaFold 3. *Nature* **2024**, *630* (8016), 493–500. https://doi.org/10.1038/s41586-024-07487-w.

(2) Pettersen, E. F.; Goddard, T. D.; Huang, C. C.; Couch, G. S.; Greenblatt, D. M.; Meng, E. C.; Ferrin, T. E. UCSF Chimera--a Visualization System for Exploratory Research and Analysis. *J. Comput. Chem.* **2004**, *25* (13), 1605–1612. https://doi.org/10.1002/jcc.20084.
